# Polyfunctional CD4^+^ T Cells As Targets for Tuberculosis Vaccination

**DOI:** 10.3389/fimmu.2017.01262

**Published:** 2017-10-05

**Authors:** Deborah A. Lewinsohn, David M. Lewinsohn, Thomas J. Scriba

**Affiliations:** ^1^Division of Infectious Disease, Department of Pediatrics, Oregon Health and Science University, Portland, OR, United States; ^2^Pulmonary and Critical Care Medicine, Department of Medicine, Oregon Health and Science University, Portland, OR, United States; ^3^Department of Medicine, VA Portland Health Care System, Portland, OR, United States; ^4^South African Tuberculosis Vaccine Initiative (SATVI), Institute of Infectious Disease and Molecular Medicine (IDM) and Division of Immunology, Department of Pathology, University of Cape Town, Cape Town, South Africa

**Keywords:** T-cell immunity, CD4^+^ T cells, vaccine-induced immunity, tuberculosis, vaccine, protective immunity, BCG, polyfunctional T cells

## Abstract

Tuberculosis (TB), caused by *Mycobacterium tuberculosis* (Mtb), remains a leading cause of morbidity and mortality worldwide, despite the widespread use of the only licensed vaccine, Bacille Calmette Guerin (BCG). Eradication of TB will require a more effective vaccine, yet evaluation of new vaccine candidates is hampered by lack of defined correlates of protection. Animal and human studies of intracellular pathogens have extensively evaluated polyfunctional CD4^+^ T cells producing multiple pro-inflammatory cytokines (IFN-γ, TNF-α, and IL-2) as a possible correlate of protection from infection and disease. In this study, we review the published literature that evaluates whether or not BCG and/or novel TB vaccine candidates induce polyfunctional CD4^+^ T cells and if these T cell responses correlate with vaccine-mediated protection. Ample evidence suggests that BCG and several novel vaccine candidates evaluated in animal models and humans induce polyfunctional CD4^+^ T cells. However, while a number of studies utilizing the mouse TB model support that polyfunctional CD4^+^ T cells are associated with vaccine-induced protection, other studies in mouse and human infants demonstrate no correlation between these T cell responses and protection. We conclude that induction of polyfunctional CD4^+^ T cells is certainly not sufficient and may not even be necessary to mediate protection and suggest that other functional attributes, such as additional effector functions, T cell differentiation state, tissue homing potential, or long-term survival capacity of the T cell may be equally or more important to promote protection. Thus, a correlate of protection for TB vaccine development remains elusive. Future studies should address polyfunctional CD4^+^ T cells within the context of more comprehensive immunological signatures of protection that include other functions and phenotypes of T cells as well as the full spectrum of immune cells and mediators that participate in the immune response against Mtb.

## Introduction

Despite the widespread global use of the only licensed tuberculosis (TB) vaccine, Bacille Calmette Guerin (BCG), TB remains a significant cause of morbidity and mortality, with 10.4 million cases and 1.8 million deaths each year (1). The WHO End TB strategy cites that a new and more efficacious vaccine is one of the interventions required for worldwide elimination of TB by 2035 (2). Yet a new vaccine that improves upon the partial protection provided by BCG remains elusive (3). One of the significant challenges to rational TB vaccine development is lack of identified immune correlates of protection. The absence of bone fide correlates hampers both pre-clinical research as well as clinical trials (4). The Food and Drug Administration defines a correlate of protection as a laboratory parameter, which is associated with protection from the occurrence of clinical disease as shown after sufficient and controlled trials (5). Correlates of protection can be further divided into ones that are causally responsible for protection (mechanistic correlates of protection) or ones that are significantly correlated with, though not the cause of protection (non-mechanistic correlates of protection) (6). The search for correlates of protection in the TB vaccine field has generally focused on mechanistic correlates and in this regard, polyfunctional CD4^+^ T cells, defined by the simultaneous co-expression of multiple pro-inflammatory cytokines (e.g., IFN-γ, TNF-α, IL-2) on a single cell level, have garnered much attention (4). While both polyfunctional CD4^+^ and CD8^+^ T cells have been defined, the TB vaccine literature investigating polyfunctional T cells as a possible correlate of protection, has focused almost exclusively on CD4^+^ T cells. Therefore, we limited our review to articles that referred to induction of IFN-γ^+^TNF-α^+^IL-2^+^ polyfunctional CD4^+^ T cells in the context of TB vaccination and vaccine immunogenicity in pre-clinical and clinical studies and focused on reviewing the published evidence that polyfunctional CD4^+^ T cells represent a mechanistic correlate of protection for TB vaccines.

With advances in multi-parameter flow cytometry to include seven or more parameters, in combination with intracellular cytokine staining (ICS) techniques, it became possible to evaluate the production of multiple cytokines simultaneously on a single cell basis. Polyfunctional CD4^+^ T cells were defined as those cells producing two or more cytokines, and were first defined within the context of examination of vaccine-induced T cell responses (7). De Rosa et al. showed that vaccines to Hepatitis B virus, tetanus, and HIV induced antigen-specific T cell responses, which were functionally complex, differed from the antigen-specific T cell responses elicited by natural infection, and were underestimated when measured by IFN-γ production alone. Furthermore, Bansal et al. demonstrated with an HIV vaccine that vaccines can elicit a more diverse array of T cells than natural infection and that vaccine dose and route can alter the cytokine profile and polyfunctionality of the T cells elicited (8). Furthermore, by varying the administration of the same vaccine, these authors illustrated that polyfunctional T cells possess a dynamic range that could be evaluated for correlates of protection in vaccine models.

Darrah et al. provided the first definitive evidence that the magnitude of the vaccine-induced polyfunctional CD4^+^ T-cell response was highly correlated with protection from infection (9). Using a mouse model of *Leishmania* disease, various vaccines induced CD4^+^ T cells displaying distinct cytokine profiles and different degrees of protection against disease upon *L. major* challenge. In this study, frequencies of MML-specific polyfunctional CD4^+^ T cells co-expressing IFN-γ, TNF-α, and IL-2 were correlated closely with the various degrees of protection elicited by a panel of vaccines. By comparison, the total number of IFN-γ-producing CD4^+^ T cells, CD4^+^ T cells producing IL-4 or IL-13, or the T regulatory cell response did not correlate with vaccine-induced protection. This study was also the first to show that BCG elicits polyfunctional CD4^+^ T cells in both the murine TB model, in which BCG mediates a degree of control of bacterial replication after *Mycobacterium tuberculosis* (Mtb) challenge, and in humans (9), as discussed further below.

In humans, polyfunctional CD4^+^ T cells have been studied with reference to severity of disease due to some intracellular infections [reviewed in Ref. (10)]. For example, slower progression to AIDS with HIV-2 than HIV-1 infection (11) and control of HIV-1 without anti-retroviral medications (12) are associated with high frequency polyfunctional HIV Gag-specific CD4^+^ T cells. By comparison, studies of polyfunctional CD4^+^ T cells in relationship to host containment of Mtb infection are contradictory. On the one hand, stronger mycobacteria-specific polyfunctional CD4^+^ T cell responses are found in adults with sputum smears negative for acid fast bacilli (AFB) than those with AFB smear positive TB (13), and in adults with latent Mtb infection (LTBI) than in those with TB (14, 15). Moreover, successful TB treatment, which rapidly reduces the bacterial load, is associated with marked increases in proportions of polyfunctional CD4^+^ T cells (13). On the other hand, other studies demonstrate that polyfunctional CD4^+^ T cell responses positively correlate with increased bacillary load. For example, there are also studies that showed stronger mycobacteria-specific polyfunctional CD4^+^ T cell responses in adults with TB than those with LTBI (16, 17) and in adults with TB than in those in healthy household contacts of adults with TB (18). These contradictory results highlight an important limitation of such correlative studies, which is that it is not possible to discern whether or not polyfunctionality of CD4^+^ T cells plays a causal role in immune control of the pathogen, or simply reflects the underlying bacterial burden.

The mechanism(s) by which polyfunctional CD4^+^ T cells induced by vaccines or natural infection may be associated with protection from infection and/or disease have not been defined. It is certainly conceivable that cells expressing multiple effector functions may be more effective in controlling infection than those producing a single pro-inflammatory cytokine. For example, IFN-γ and TNF-α act synergistically to enhance the ability of macrophages to contain *L. major* infection (19, 20), which in turn is associated with enhanced control of disease by the combination of IFN-γ and TNF-α in the murine model (20). Similarly, IFN-γ and TNF-α synergistically inhibit Mtb replication within murine macrophage cell lines (21). As first defined in the murine *Leishmania* model, among vaccine-induced CD4^+^ T cells producing IFN-γ, TNF-α, and/or IL-2, cells producing all three cytokines (3^+^ cells) produce more cytokine on a per cell basis [as defined by mean fluorescence intensity (MFI)], than do those that produce two cytokines (2^+^ cells), which in turn produce more cytokine than cells producing a single cytokine (1^+^ cells) (9). Moreover, Darrah et al. defined integrated MFI (iMFI), a metric that combines the frequency of each cytokine-producing CD4^+^ T cell response with its associated MFI and showed that the iMFI for each of IFN-γ, TNF-α, or IL-2, also correlated with the degree of vaccine-induced protection. This analysis is consistent with the interpretation that it may be the high potency of multifunctional T cells to produce cytokines on a per cell basis that is associated with vaccine-induced protection. Finally, Seder et al. proposed a linear differentiation model for CD4^+^ T cells based upon their cytokine profile (22). In this model, naïve CD4^+^ T cells, upon activation with antigen, first acquire the capacity to secrete TNF-α or IL-2, followed by the capacity to produce both cytokines and finally, upon further differentiation, additionally produce IFN-γ as well. These early lineage T cells also express CCR7, consistent with central memory T cells (T_CM_ cells). Subsequently, with continued antigenic stimulation these polyfunctional T cells lose CCR7 expression and capacity to secrete TNF-α or IL-2, and finally produce only IFN-γ in the highly differentiated, terminal effector stage. Thus, according to this model, protective potential of a polyfunctional CD4^+^ T cell producing IFN-γ, TNF-α, and IL-2 may be associated with its degree of differentiation and simultaneous capacity for memory and for effector function.

## Polyfunctional T Cells as a Biologically Plausible Candidate for a Mechanistic Correlate of Protection in TB

The literature regarding the possible role of polyfunctional CD4^+^ T cells in mediating vaccine-induced protection for TB has primarily investigated those cells co-producing IFN-γ, TNF-α, and IL-2, hereafter, we will refer to as “polyfunctional CD4^+^ T cells.” There is substantial evidence derived from study of murine TB models and humans that all three of these cytokines are necessary for the control of Mtb infection.

Essential roles in host defense for IFN-γ, and for CD4^+^ T cells that produce IFN-γ, were initially defined in the murine TB model using IFN-γ-deficient mice (23, 24), CD4^+^ T cell deficient mice, and adoptive transfer of CD4^+^ T cells in the murine TB model [reviewed in Ref. (25)]. More recently, Green et al. showed that CD4^+^ T cells are important as a source of IFN-γ mediating protection from Mtb, by demonstrating that IFN-γ production from all other cellular sources than CD4^+^ T cells was insufficient in controlling chronic infection and maintaining survival and that CD4^+^ T cell derived IFN-γ promoted CD8^+^ T cell responses (26). Consistent with this, humans deficient in IL-12 receptor expression (27), IFN-γ receptor expression, or IFN-γ signaling (28) are more susceptible to mycobacterial disease. However, it is important to note that these deficiencies are not T cell-specific and thus the precise role of T cell-derived IFN-γ vs other sources of IFN-γ in humans remains unresolved. At least in the murine TB model, a mechanism by which IFN-γ may mediate its effector function is through activation of macrophages, which in turn inhibit Mtb growth *via* induction of iNOS and autophagy [reviewed in Ref. (29)].

TNF-α is also essential for TB host defense. Mice deficient in TNF receptor or TNF-α are highly susceptible to Mtb infection (30, 31). There are multiple cell types that produce TNF-α, including innate immune cells, epithelial cells, endothelial cells, and fibroblasts. Early control of Mtb infection in the mouse model, prior to the acquisition of adaptive immunity, is primarily mediated by TNF-α derived from macrophages (32). Conversely, mice deficient for TNF-α expression in T cells poorly control chronic infection (32). In humans, pharmacological blockade of TNF-α, used for treatment of rheumatologic disorders, increases susceptibility to mycobacterial disease (33, 34). As demonstrated in both the murine TB model and in humans, TNF-α promotes the formation of mature granulomas and like IFN-γ, also activates infected macrophages, which, at least in mice, contain infection *via* induction of iNOS and autophagy [reviewed in Ref. (29)].

IL-2 induces proliferation, promotes the survival of TCR-activated T cells [reviewed in Ref. (35)] and promotes the development of fully competent memory T cells during primary infection (36). Therefore, IL-2 is generally assumed to aid TB host defense through supporting the expansion and maintenance of the T cell response [reviewed in Ref. (35)]. IL-2 also has a role in T cell tolerance, through its role in maintaining CD4^+^CD25^+^ regulatory T cells. In support of this, IL-2 and IL-2R deficient mice develop early severe autoimmune disease [reviewed in Ref. (35)]. Consequently, these mice cannot be utilized to determine the role of IL-2 in TB host defense as has been done with IFN-γ and TNF-α. Nonetheless, in the murine TB model, induction of IL-2-producing CD4^+^ T cells is associated with vaccine-induced protection to Mtb and loss of IL-2-producing CD4^+^ T cells is associated with loss of protection [reviewed in Ref. (37)]. In humans, decreased proportions of polyfunctional CD4^+^ T cells in individuals with AFB smear positive TB compared to those with AFB smear negative TB, or in those with TB disease as compared to those with LTBI, was associated with decreases in proportions of total IL-2 producing T cells (13, 14).

## TB Vaccines Induce Polyfunctional T Cells in Animal Models

BCG and novel TB vaccine candidates utilizing various antigens and vaccine platforms induce polyfunctional CD4^+^ T cells in murine, bovine, and non-human primate (NHP) TB models. These T cell populations possess attributes, which are critical for mediating vaccine-mediated immunity in that they can traffic to the lung and include memory T cell populations that persist in the vaccinated host.

### BCG and Other Live Mycobacterial Vaccines Elicit Polyfunctional CD4^+^ T Cells

As the only licensed TB vaccine and in light of its demonstrated record of partial efficacy, BCG is often used as a control vaccine in the mouse model of TB vaccination (Table 1). Several studies have shown that BCG induces polyfunctional mycobacteria-specific CD4^+^ T cells detected among lung (9, 38) and splenic (9, 39–44) lymphocyte populations (Table 1). Darrah et al. found that polyfunctional mycobacterial CD4^+^ T cells produced more cytokine on a per cell basis than do those producing two or one cytokines, similar to *Leishmania*-specific polyfunctional CD4^+^ T cells (9). In the murine TB model, these T cell responses constitute minor (38, 39, 42, 44), predominant (43), or major (9) subsets within the first 2–4 months after immunization. In addition, polyfunctional CD4^+^ T cells have been detected among CD44^+^ memory T cell populations as early as 3 weeks after vaccination (40) and persist as a major subset more than 40 weeks after vaccination (38). By contrast, though detecting polyfunctional CD4^+^ T cells as a predominant T cell population 2 and 8 months after BCG, Derrick et al. were unable to detect these cells 14 months after immunization, suggesting that this subset may not persist long-term (43). These differences in persistence of polyfunctional CD4^+^ T cells and wide phenotypic spectrum in cytokine-producing CD4^+^ T cells are not readily reconciled between studies. However, differences in the experimental protocols utilized, such as differences in BCG strain and/or delivery method, mouse strain and/or cell populations studied and the nature and dose of the Mtb challenge, may underlie the inconsistency in findings. Finally, BCG induces polyfunctional T cells in cattle (45) and rhesus macaque monkeys of Chinese origin (*Macaca mulatta*) (46). Maggioli et al. showed that BCG induced mycobacteria-specific polyfunctional CD4^+^ T cells as a major cytokine expressing subset in peripheral blood mononuclear cells (PBMC) obtained from calves 6 weeks after BCG and cultured with antigen for 2 weeks *in vitro* (45). These T cells expressed phenotypic markers consistent with memory T cells. Finally, White et al. showed that BCG delivered by the aerosol route induced PPD-specific polyfunctional CD4^+^ T cells as a major subset in both PBMC and bronchoalveolar lavage (BAL), fluid of Chinese rhesus macaque monkeys 8 weeks after BCG administration (46).

**Table 1:** BCG induces polyfunctional CD4^+^ T cells in animal models of TB.

BCG strain	Study design	IA	Conclusions	Reference
SSI	C57BL/6 mice	*Cell measured*: CD4^+^ 3^+^ cells	BCG induced a major subset in lung and spleen at 4 months	Darrah et al. (9)
	BCG i.m. (+0)	*Cellular source*: spleen and lung		
	*IA*: (+4 months)	*Antigen(s)*: PPD		

China	C57BL/6 mice	*Cell measured*: CD4^+^ 3^+^ cells	BCG induced a minor subset in spleen at 12 and 32 weeks	Yuan et al. (44)
	BCG s.c. (+0)	*Cellular source*: spleen		
	*IA*: (+12, +32 weeks)	*Antigen(s)*: Ag85B (p.p.); HspX (p.p.); PPD		

SSI	Balb/c mice	*Cell measured*: CD4^+^ 3^+^ cells	BCG induced a minor subset in spleen at 13 and 22 weeks	Tchilian et al. (42)
	BCG s.c. (+0)	*Cellular source*: spleen		
	*IA*: (+13, +22 weeks)	*Antigen(s)*: PPD; Ag85A (p.p.)		

Pasteur	C57BL/6 mice	*Cell measured*: CD4^+^ 3^+^ cells	BCG induced a predominant subset in spleen at 2 and 8 months, not detected at 14 months	Derrick et al. (43)
	BCG s.c. (+0)	*Cellular source*: spleen		
	*IA*: (+2, +8, +14 months)	*Antigen(s)*: BCG		

Pasteur	Balb/c mice	*Cell measured*: CD4^+^ 3^+^ cells	BCG induced a minor subset in spleen at 14 weeks	Forbes et al. (39)
	BCG s.c. (+0)	*Cellular source*: spleen and lung		
	*IA*: (+14 weeks)	*Antigen(s)*: PPD; Ag85A (p.p.)		

SSI	CB6F1	*Cell measured*: CD4^+^CD44^hi^ 3^+^ cells	BCG induced TB10.4- but not Ag85B-specific memory T cells in spleen after 3 weeks	Elvang et al. (40)
	BCG s.c. (+0)	*Cellular source*: spleen		
	*IA*: (+3 weeks)	*Antigen(s)*: rTB10.4; rAg85B		

SSI	C57BL/6 mice	*Cell measured*: CD4^+^CD44^hi^ 3^+^ cells	BCG induced a minor subset of memory T cells in lung and spleen at 10 weeks	Lindenstrom et al. (38)
	BCG s.c. (+0)	*Cellular source*: lung and spleen		
	*IA*: (+10 weeks)	*Antigen(s)*: PPD		

SSI	C57BL/6 mice	*Cell measured*: CD4^+^CD44^hi^ 3^+^ cells	BCG induced a major subset of memory T cells in spleen after 40 weeks	Lindenstrom et al. (41)
	BCG s.c. (+0)	*Cellular source*: spleen		
	*IA*: (>40 weeks)	*Antigen(s)*: PPD		

SSI	Holstein steers	*Cell measured*: CD45RO^+^ CCR7^+^ CD4^+^ 3^+^ cells after 2 weeks *in vitro* culture	After 2 weeks of *in vitro* culture, a major subset of memory T cells was identified 6 weeks after BCG	Maggioli et al. (45)
	BCG s.c. (+0)	*Cellular source*: PBMC		
	*IA*: (+6 weeks)	*Antigen(s)*: rAg85A; rTB10.4		

SSI	*Macaca mulatta*	*Cell measured*: CD4^+^ 3^+^ cells	BCG induced a major subset of T cells in PBMC and lung (BAL) after 8 weeks	White et al. (46)
	BCG aerosol (+0)	*Cellular source*: PBMC; BAL		
	*IA*: (+8 weeks)	*Antigen(s)*: PPD		

*SSI, Staten Serum Institute; i.m., intramuscular; s.c., subcutaneous; i.d., intradermal; CB6F1, C57BL/6 X Balb/c; 3^+^, IFN-γ^+^TNF-α^+^IL-2^+^; PBMC, peripheral blood mononuclear cells; BAL, bronchoalveolar lavage; p.p., peptide pool; BCG, Bacille Calmette Guerin; TB, tuberculosis; IA, immune assay*.

*Major subset = subset that constitutes >50% of the total cytokine-producing cells; minor subset = subset that constitutes <20% of the total cytokine-producing cells; predominant subset = subset that constitutes 20–50% of the total cytokine-producing cells*.

Similarly, recombinant BCG constructs (42, 44) induce polyfunctional CD4^+^ T cell responses in mice (Table 2). Yuan et al. compared BCG overexpressing Ag85B and HspX (rBCG:XB) to non-recombinant BCG and demonstrated that rBCG:XB elicited comparable PPD- and Ag85B-specific and stronger HspX-specific polyfunctional T cell responses 12 and 32 weeks after vaccination (44). Comparing a recombinant BCG expressing membrane-perforating listerolysin (with the rationale of promoting CD8^+^ T cell responses from cytosolic antigens) and deficient in urease C (ΔureC *hly*^+^, VPM1002), Tchilian et al. demonstrated low-frequency splenic PPD- and Ag85A-specific polyfunctional CD4^+^ T cells, comparable to those induced by BCG, 13 and 22 weeks after immunization (42). Maggioli et al. showed that vaccination with a cocktail of four distinct BCG deletion mutants (Δfdr8, ΔleuCD, Δpks16, ΔmmaA2, and ΔmetA) induced mycobacteria-specific polyfunctional CD4^+^ T cells in 2 weeks cultures of PBMC from bovine calves 6 weeks after vaccination. These responses were comparable to those induced by non-recombinant BCG (45).

**Table 2:** TB vaccines induce polyfunctional CD4^+^ T cells in animal models of TB.

TB vaccine	Study design	IA	3^+^ cells/protection^a^	Conclusions	Reference
BCG (SSI)	*Mouse strain*: C57BL/6 *Immunization*: BCG i.m: (+0) *Challenge*: Mtb Erdman aerosol (+3 months) *Post-challenge*: lung and spleen CFU (+1 month)	*Cell measured*: CD4^+^ 3^+^ cells *Cellular source*: spleen and lung *Antigen(s)*: PPD *Pre-challenge*: (+4 months)	+ BCG induced 3^+^ memory T cells and protection in lung and spleen as compared to naïve mice	BCG induced a major subset in lung and spleen at 4 months. Correlation of pre-challenge 3^+^ T cell frequencies with protection with BCG vs naïve mice	Darrah et al. (9)

rBCG:XB BCG (China)	*Mouse strain*: C57BL/6 mice *Immunization*: rBCG:XB s.c. vs BCG s.c: (+0) *Challenge*: Mtb H37Rv i.n. (+12 weeks) *Post-challenge*: lung and spleen CFU (+4, +10, +20 weeks)	*Cell measured*: CD4^+^ 3^+^ cells *Cellular source*: spleen *Antigen(s)*: Ag85 (p.p.); HspX (p.p.); PPD *Pre-challenge*: (+12, +32 weeks)	++ rBCG:XB vs BCG: 1 log protection in spleen and lung	rBCG:XB induced more HspX-specific 3^+^ T cells than BCG, which correlated with greater protection in lung and spleen	Yuan et al. (44)

VPM1002 BCG (SSI) MVA85A	*Mouse strain*: Balb/c mice *Immunization*: BCG s.c. (+0) vs VPM1002 s.c. (+0) vs BCG s.c. (+0)/MVA85A i.d. (+10 weeks) vs VPM1002 s.c. (+0)/MVA85A i.d. (+10 weeks) *Challenge*: Mtb H37Rv aerosol (+14 weeks) *Post-challenge*: lung and Spleen CFU (+12 weeks)	*Cell measured*: CD4^+^ 3^+^ cells *Cellular source*: spleen *Antigen(s)*: PPD; Ag85A (p.p.) *Pre-challenge*: (+13, +22 weeks)	– *3^+^ T cell response*: BCG/MVA85A > VPM1002/MVA85A >> BCG or VPM1002 *Protection*: VPM1002 or VPM1002/MVA85A > BCG or BCG/MVA85A	BCG and VPM1002 induced a minor subset of 3^+^ T cells. MVA85A boost to either BCG or VPM1002 induced 3^+^ T cells. The 3^+^ T cell response did not correlate with protection	Tchilian et al. (42)

E6-85: DDA/MPL AdE6-85 pVax6-85 BCG (Pasteur)	*Mouse strain*: C57BL/6 *Immunization*: E6-85 in DDA/MPL s.c. (+0, +2, +4 weeks) vs AdE6-85 i.m. (+0, +1 month) vs pVaxE6-85 i.m. (+0, +3, +6 weeks) vs BCG s.c. (+0); vs BCG mixed with E6-85 in DDA/MPL s.c. (+0, +2, +4 weeks) *Challenge*: Mtb Erdman aerosol (+2, +8, +14 months) *Post-challenge*: lung and spleen CFU (+1 month)	*Cell measured*: CD4^+^ 3^+^ cells *Cellular source*: spleen *Antigen(s)*: BCG *Pre-challenge*: (+2, +8, +14 months)	+++ The hierarchy of 3^+^ T cell frequencies correlated with that of protection in lung > spleen at 14 months	Strong correlation between the 3^+^ T cell frequency and degree of protection induced by several vaccine regimens	Derrick et al. (43)

BCG (Pasteur) Ad85A	*Mouse strain*: Balb/c *Immunization*: BCG s.c. (+0)/Ad85A i.d. (+10 weeks); vs BCG s.c. (+0)/Ad85A i.n. (+10 weeks) *Challenge*: Mtb Erdman aerosol (+14 weeks) *Post-challenge*: lung and spleen CFU (+6 weeks)	*Cell measured*: CD4^+^ 3^+^ cells *Cellular source*: spleen and lung *Antigen(s)*: PPD; Ag85A (p.p.) *Pre-challenge*: (+14 weeks)	+/− Ad85A i.n. boost induced more 3^+^ T cells and more protection in lung than Ad85A i.d. boost. However, Ad85A i.d. boost induced more 3^+^ T cells in spleen than Ad85A boost	Boosting i.d. but not i.n. induced Ag85A-specific 3^+^ T cells as a predominant subset in spleen. Boosting i.n. but not i.d. induced Ag85A-specific 3^+^ T cells in lung. Lung, not splenic 3^+^ T cells correlated with protection in the lung	Forbes et al. (39)

H4:CAF01 Ad-H4 BCG (SSI)	*Mouse strain*: CB6F1 *Immunization*: Ad-H4 s.c. (+0) vs H4 in CAF01 s.c. (+0, +2 weeks) vs H4 in CAF01 s.c. (+0)/Ad-H4 (+2 weeks) vs BCG s.c. (+0) *Challenge*: Mtb Erdman aerosol (+8 weeks) *Post-challenge*: lung and spleen CFU (+6 weeks)	*Cell measured*: CD4^+^CD44^hi^ 3^+^ cells *Cellular source*: spleen *Antigen(s)*: TB10.4 (p.p.); Ag85B (p.p.) *Pre-challenge*: (+3 weeks)	++ H4:CAF01/Ad-H4 induced more memory 3^+^ CD4^+^ T cells, and was more protective in lung than H4:CAF01 or Ad-H4 alone	Correlation between the 3^+^ T cell frequency and degree of protection induced by three different vaccine regimens	Elvang et al. (40)

VSV-836 BCG (SSI)	*Mouse strain*: Balb/c *Immunization*: VSV i.n. (+0) vs BCG i.m. (+0) vs BCG i.m. (+0)/VSV i.n. (+12 weeks)	*Cell measured*: CD4^+^ 3^+^ cells *Cellular source*: spleen *Antigen(s)*: TFP846 *Immunogenicity*: (+18 weeks)	N/A	Neither VSV nor BCG/VSV induced 3^+^ T cells	Zhang et al. (49)

rAg85B:CpG rAg85B:CpG in NP	*Mouse strain*: C57BL/6 *Immunization*: rAg85B with CpG i.n. vs rAg85B with CpG i.d. vs rAg85B with CpG in NP i.n. vs rAg85B with CpG in NP i.d. (+0, +7, +21 days) *Challenge*: Mtb Erdman aerosol (+49 days) *Post-challenge*: lung CFU (+1 month)	*Cell measured*: CD4^+^ 3^+^ cells *Cellular source*: spleen *Antigen(s)*: rAg85B *Pre-challenge*: (+28, +49 days)	++ rAg85B/CpG/NP i.n. induced more 3^+^ T cells and more protection in lung than did rAg85B/CpG	rAg85B in CpG ± NP induced 3^+^ T cells as a predominant subset Correlation between the 3^+^ T cell frequency and degree of protection induced by two different vaccine regimens	Ballester et al. (50)

rMT1721:GLA DNA-MT1721	*Mouse strain*: C57BL/6 *Immunization*: rMT1721 in GLA s.c (0)/DNA-MT1721 i.m. (+4, +8 weeks) vs DNA-MT1721 i.m. (0)/rMT1721 in GLA s.c (+4, +8 weeks)	*Cell measured*: CD4^+^ 3^+^ cells *Cellular source*: spleen *Antigen(s)*: rMT1721 *Immunogenicity*: (+10 weeks)	N/A	rMT1721 as either a prime or a boost to DNA-MT1721 induced 3^+^ T cells as a minor subset in splenocytes	Cayabyab et al. (51)

H4:IC31	*Mouse strain*: CB6F1 *Immunization*: H4 in IC31 s.c. (+0, +2, +4 weeks) comparing 0.5 μg vs 5 μg *Challenge*: Mtb Erdman aerosol (+10 weeks) *Post-challenge*: lung CFU (+6 weeks)	*Cell measured*: CD4^+^ 3^+^ cells *Cellular source*: PBMC *Antigen(s)*: rAg85B; rTB10.4; H4 *Pre-challenge*: (+5 weeks)	++ H4:IC31 induced stronger 3^+^ T cells at a lower dose (0.5 μg) vs a higher dose (5.0 μg), which correlated with better protection in the lung	H4:IC31 induced 3^+^ T cells, as a major subset in PBMC Correlation of 3^+^ T cell frequencies with protection with H4:IC31 delivered at lower vs higher doses	Aagaard et al. (52)

H1:CAF01 BCG (SSI)	*Mouse strain*: C57BL/6 *Immunization*: BCG s.c. (+0) vs BCG s.c. (+0)/H1 in CAF01 s.c. (+4 weeks) *Challenge*: Mtb Erdman aerosol (+10 weeks) *Post-challenge*: lung CFU (+7, +26, +50 weeks)	*Cell measured*: CD4^+^CD44^hi^ 3^+^ cells *Cellular source*: spleen and lung *Antigen(s)*: PPD *Pre-challenge*: (+10 weeks)	+ BCG/H1:CAF01 induced more memory 3^+^ memory T cells and protection in lungs as compared to naïve mice	BCG/H1:CAF01 and BCG induced a minor subset of memory 3^+^ T cells in lung and spleen Correlation of pre-challenge 3^+^ T cell frequencies with protection with BCG/H1:CAF01 vs naïve mice	Lindenstrom et al. (38)

H1:CAF01 BCG (SSI)	*Mouse strain*: C57BL/6 *Immunization*: BCG s.c. (+0) vs H1 in CAF01 s.c. (+0, +2, +4 weeks) *Challenge*: Mtb Erdman aerosol (+14 months) *Post-challenge*: lung CFU (+6 weeks)	*Cell measured*: CD4^+^CD44^hi^ 3^+^ cells *Cellular source*: spleen *Antigen(s)*: H1; PPD *Pre-challenge*: (+52 weeks)	+ H1:CAF01 and BCG induced comparable levels of 3^+^ memory CD4^+^ T cells and protection in lung as compared to naïve mice.	H1:CAF01 induced a major subset of memory 3^+^ T cells after 52 weeks Correlation of pre-challenge 3^+^ T cell frequencies with protection with H1:CAF01 or BCG vs naïve mice.	Lindenstrom et al. (41)

H1:MMG/DDA H1:M_32_MMG/DDA	*Mouse strain*: C57BL/6 *Immunization*: H1 in MMG/DDA s.c. (+0, +2, +4 weeks) vs H1 in M_32_MMG/DDA s.c. (+0, +2, +4 weeks)	*Cell measured*: CD4^+^ 3^+^ cells *Cellular source*: spleen *Antigen(s)*: H1 *Immunogenicity*: (+6 weeks)	NA	H1:MMG/DDA or H1:M_32_MMG/DDA induced 3^+^ T cells as a major subset in splenocytes	Andersen et al. (53)

ID93:GLA-SE	*Mouse strain*: C57BL/6 *Immunization*: ID93 in GLA-SE s.c. (+0, +3, +6 weeks) *Challenge*: Mtb H37Rv aerosol (+9–10 weeks) *Post-challenge*: lung and spleen CFU (+4 weeks)	*Cell measured*: CD4^+^ CD44^+^3^+^ cells *Cellular source*: spleen *Antigen(s)*: ID93 *Pre-challenge*: (+9 weeks)	+ ID93:GLA-SE induced more memory 3^+^ memory T cells and protection in lungs as compared to control mice	ID93:GLA-SE induced memory 3^+^ T cells as a minor subset in splenocytes, which correlated with protection in the lung vs control mice	Bertholet et al. (54)

BCG mutant cocktail (SSI)	*Holstein steers*: newborn calves *Immunization*: BCG mutant cocktail s.c. (+0)	*Cell measured*: CD45RO^+^ CCR7^+^ CD4^+^ 3^+^ cells after 2 weeks *in vitro* culture *Cellular source*: PBMC *Antigen(s)*: rAg85A; rTB10.4 *Immunogenicity*: (+6 weeks)	N/A	After 2 weeks of *in vitro* culture, a major subset of memory T cells was identified 6 weeks after BCG mutants	Maggioli et al. (45)

BCG (SSI) AERAS-401 AERAS-402	*Monkeys*: *Macaca mulatta* *Immunization*: BCG i.d. (+0)/AERAS-402 i.m. (+15, +27 weeks) vs AERAS-401 i.d. (+0)/AERAS-402 i.m. (+15, +27 weeks)	*Cell measured*: CD4^+^ 3^+^ cells *Cellular source*: PBMC *Antigen(s)*: Ag85A (p.p.); Ag85B (p.p.); TB10.4 (p.p.) *Immunogenicity*: (+1, +4, +8, +15, +16, +19, +20, +24, +27, +28, +31, +37 weeks)	N/A	AERAS-402 delivered as a boost to either BCG or AERAS-401 elicited transient 3^+^ T cell responses in PBMC 1 week after the first boost	Magalhaes et al. (47)

AERAS-402	*Monkeys*: *M. mulatta* *Immunization*: AERAS-402 *via* aerosol (+1, +8, +15 days)	*Cell measured*: CD4^+^ 3^+^ cells *Cellular source*: PBMC and BAL *Antigen(s)*: Ag85A/B (p.p.) *Immunogenicity*: (+18, +43 days)	N/A	AERAS-402 delivered *via* aerosol induced weak, detectable 3^+^ T cells in PBMC 3 days and in BAL 28 days after completing immunization	Hokey et al. (48)

*SSI, Staten Serum Institute; rBCG:XB, Recombinant BCG overexpressing Ag85B and HspX; VPM1002, Recombinant BCG which is urease C deficient and expressing membrane-perforating listerolysin (*L. monocytogenes*); MVA85A, Modified Vaccinia Ankara expressing Ag85A; E6-85, recombinant ESAT-6 fused to the n-terminus of Ag85B without the signal sequence; DDA, dimethyl dioctadecyl ammonium bromide; MPL, monophosphoryl lipid A; AdE6-85, adenoviral vector (Ad5) expressing E6-85; pVax6-85, DNA construct expressing E6-85; Ad85A, human adenoviral vector expressing Ag85A; H4, Recombinant Ag85B/TB10.4 fusion protein; CAF01, cationic liposomes formulated with synthetic mycobacterial cord factor; Ad-H4, Adenoviral (Ad5) vector expressing H4; VSV-836, Vesicular Stomatitis Virus (VSV) expressing a fusion of Rv3615c, Mtb10.4, Rv2660c (TFP846); rAg85B, recombinant Ag85B; NP, polypropylene sulfide nanoparticles; rMT1721, recombinant MT1721; GLA, glucopyranosyl lipid A; IC31, cationic peptide and oligodeoxynucleotide (ODN1); H1, Recombinant Ag85B/ESAT-6 fusion protein; MMG, monomycoloyl glycerol: M_32_MMG, synthetic analog of MMG; ID93, Recombinant Rv2608, Rv3619, Rv3620, and Rv1813 fusion protein; GLA-SE, synthetic MPL in stable oil-in-water nanoemulsion; BCG mutant cocktail, BCG Δfdr8, ΔleuCD, Δpks16, ΔmmaA2, and ΔmetA; AERAS-401, Recombinant BCG expressing perfringolysin; AERAS-402, Adenoviral vector (Ad35) expressing Ag85A, Ag85B, and TB10.4; CB6F1, C57BL/6 X Balb/c; i.m., intramuscular; s.c., subcutaneous; i.d., intradermal; i.n., intranasal; CFU, colony forming units; 3^+^, IFN-γ^+^TNF-α^+^IL-2^+^; PBMC, peripheral blood mononuclear cells; BAL, bronchoalveolar lavage; p.p., peptide pool; BCG, Bacille Calmette Guerin; TB, tuberculosis, Mtb, *M. tuberculosis*; IA, immune assay*.

*+ = weak evidence for 3^+^ cells as a correlate of protection (positive correlation between 3^+^ T cell frequency and one or two similarly performing vaccines vs control); ++ = moderate evidence for 3^+^ cells as a correlate of protection (positive correlation between 3^+^ T cell frequency and at least two differently performing vaccines ± control); – = evidence against 3^+^ cells as a correlate of protection (no correlation between 3^+^ T cell frequency and four vaccines); +++ = strongest evidence for 3^+^ cells as a correlate of protection (positive correlation between 3^+^ T cell frequency and five vaccines); +/− = equivocal evidence for 3^+^ cells as a correlate of protection; N/A, Not applicable*.

*Major subset = subset that constitutes >50% of the total cytokine-producing cells; minor subset = subset that constitutes <20% of the total cytokine-producing cells; predominant subset = subset that constitutes 20–50% of the total cytokine-producing cells*.

*^a^Vaccine-induced 3^+^ T cells correlate with protection from Mtb challenge. Protection is defined as control of Mtb replication*.

### Vaccine Candidates Utilizing Viral Vectors Can Elicit Polyfunctional CD4^+^ T Cells

Adenoviral vectors expressing Mtb proteins also elicit polyfunctional CD4^+^ T cells in murine and NHP models of TB (Table 2). Derrick et al. showed that a replication deficient adenoviral vector, Ad5, expressing ESAT-6 fused to the n-terminus of Ag85B (E6-85) induced BCG-specific polyfunctional CD4^+^ T cells, detected in spleen of mice 2 and 8 months after vaccination, but no longer detectable 14 months after vaccination (43). Forbes et al. used an Ad5 vector expressing Ag85A as a boost to BCG and compared intradermal (i.d.) to intranasal (i.n.) delivery of the adenovirus (39). Boosting i.d. but not i.n. induced Ag85A-specific polyfunctional CD4^+^ T cells, as a predominant subset in spleen 4 weeks after the adenoviral boost vaccination. By contrast, when lung T cells were examined, this adenoviral vector only induced Ag85A-specific polyfunctional CD4^+^ T cells when delivered i.n. but not i.d. Elevang et al. showed that an Ad5 vector expressing H4, a recombinant Ag85B/TB10.4 fusion protein (Ad-H4), induced Ag85A and TB10.4-specific polyfunctional CD4^+^ T cells, representing a predominant subset of CD44^+^ memory T cells in spleen 3 weeks following the vaccination and also when administered as a boost to recombinant H4 delivered in CAF01 (H4:CAF01) 1 week following this vaccination (40). Magalhaes et al. showed that an adenoviral vector, Ad35, expressing Ag85A, Ag85b, and TB10.4 (AERAS-402) delivered i.m. as a boost to either BCG or recombinant BCG expressing perfringolysin (AERAS-401), elicited transient Ag85A/B and TB10.4-specific polyfunctional CD4^+^ T cell responses in PBMC of rhesus macaque monkeys of Chinese origin (*M. mulatta*) 1 week after the first boost (47). Hokey et al. went on to show that AERAS-402 delivered *via* aerosol to Chinese rhesus macaques induced weakly detectable Ag85A/B-specific polyfunctional CD4^+^ T cells in PBMC 3 days after and in BAL 28 days after completion of immunization (48). Therefore, at least in the short-term, adenoviral vectors expressing Mtb proteins elicit polyfunctional CD4^+^ T cells when delivered with alone or as a boost to BCG in mice and NHPs.

Two additional viral vector systems have also been assessed in the mouse TB model (Table 2). A vesicular stomatitis virus (VSV) construct expressing Rv3615c, Mtb10.4, and Rv2660c elicited dual-cytokine-producing (IFN-γ^+^TNF-α^+^; TNF-α^+^IL-2^+^; and IFN-γ^+^IL-2^+^) but not triple-cytokine producing (IFN-γ^+^TNF-α^+^IL-2^+^) polyfunctional CD4^+^ T cell responses when delivered i.n. either alone or as a boost to BCG (49). By contrast, a modified vaccinia Ankara (MVA) expressing Ag85A (MVA85A), when delivered as a boost to BCG, elicited predominantly Ag85A-specific polyfunctional CD4^+^ T cells 3 and 12 weeks following MVA immunization (42). The capacity for MVA85A to elicit polyfunctional CD4^+^ T cell responses in humans has been more extensively evaluated and is discussed below.

### Recombinant Antigen Vaccines Elicit Polyfunctional CD4^+^ T Cells

Recombinant Mtb antigen subunit vaccines, typically comprising proteins formulated in adjuvant, have been extensively evaluated in the mouse TB model (Table 2). Several adjuvanted recombinant Mtb proteins or fusions of multiple Mtb proteins have been shown to elicit polyfunctional CD4^+^ T cells in mice, including recombinant Ag85B (50), and MT1721 (51) and fusion proteins comprised of ESAT-6 fused to the n-terminus of Ag85B (E6-85) (43), Ag85B/TB10.4 (H4) (40, 52), Ag85B/ESAT-6 (H1) (38, 41, 53), and gene products of Rv2608, Rv3619, Rv3620, and Rv1813 (ID93) (54). These recombinant protein vaccines have successfully utilized several different adjuvant formulations based upon immunostimulatory lipids (38, 40, 41, 43, 53, 54) or nucleotides (50–52) to elicit these T cell responses. In addition, adjuvanted recombinant protein vaccines induce polyfunctional CD4^+^ T cell responses when used alone (40, 41, 43, 50, 52–54) and when administered as a boost after BCG prime (38, 43) or a DNA vaccine prime (51). Moreover, one study examined antigen dose and found that a lower dose of H4:IC31 [recombinant H4 delivered in IC31 (cationic peptide and oligodeoxynucleotide), 0.5 μg] induced greater frequencies of polyfunctional CD4^+^ T cells than a higher dose (5 μg) (52). Finally, recombinant protein vaccines induce polyfunctional CD4^+^ T cells detected among CD44^+^ memory T cell populations (38, 40, 41) as early as 1 week following completed immunization (40) and persisted 56 weeks when recombinant H1 delivered in CAF01 (H1:CAF01) was used (41) and as long as 14 months when recombinant E6-85 delivered in DD/MPL (liposomal monophosphoryl lipid A, E6-85: DDA/MPL) was used to boost BCG (43). Therefore, polyfunctional CD4^+^ T cells are induced with several distinct adjuvanted recombinant Mtb protein vaccines, when used alone or as a boost to BCG or DNA vaccine, and are included among memory CD4^+^ T cell populations.

### TB Vaccines Promote Polyfunctional CD4^+^ T Cells in the Lung

Recent studies in the mouse TB demonstrate the importance of tissue location for protective immune responses against *M. tuberculosis* and suggest that lung resident CD4^+^ T cells mediate control of Mtb infection better than CD4^+^ T cells that reside in the pulmonary vasculature but do not enter the parenchyma [reviewed in Ref. (37)]. Protective parenchymal CD4^+^ T cells express activation markers such as PD-1 and CD69, do not express the terminal differentiation marker KLRG1, and produce less IFN-γ than intravascular T cells. Therefore, the ability of TB vaccines to promote the accumulation of lung resident polyfunctional CD4^+^ T cells may be an additional important component of vaccine-induced protection by these cells.

Tuberculosis vaccine candidates can elicit polyfunctional CD4^+^ T cells in the lung (Table 3). Darrah et al. showed that BCG delivered i.m. induced PPD-specific polyfunctional CD4^+^ T cells, as a major subset, in the lung 4 months after vaccination (9). Lindenstrom et al. showed that BCG and BCG boosted with Ag85B-ESAT-6 fusion protein in CAF01 (H1:CAF01) induced PPD-specific polyfunctional CD4^+^ T cells, as a minor subset in lung 10 weeks after the completed immunization (38). By contrast, Forbes et al. showed that an Ag85A-expressing adenoviral vaccine-induced polyfunctional CD4^+^ T cells in the lung after i.n. but not i.d. administration of the vaccine (39). In NHP’s, polyfunctional CD4^+^ T cells are detected in BAL after aerosol delivery of BCG (46) or adenoviral vaccine, AERAS-402 (48).

**Table 3:** Lung resident polyfunctional CD4^+^ T cells before and after TB vaccination.

TB vaccine	Study design	IA	Conclusions	Reference
BCG (SSI)	*Mouse strain*: C57BL/6 *Immunization*: BCG i.m: (+0)	*Cell measured*: CD4^+^ 3^+^ cells *Cellular source*: lung *Antigen(s)*: PPD *Immunogenicity*: (+4 months)	BCG induced a major subset in lung at 4 months after vaccination	Darrah et al. (9)

BCG (SSI) H1:CAF01	*Mouse strain*: C57BL/6 *Immunization*: BCG s.c. (+0) vs BCG s.c. (+0)/rH1 in CAF01 s.c. (+4 weeks) *Challenge*: Mtb Erdman aerosol (+10 weeks) *Post-Challenge*: lung CFU (+7, +26, +50 weeks)	*Cell measured*: CD4^+^CD44^hi^ 3^+^ cells *Cellular source*: lung *Antigen(s)*: PPD; Ag85A, ESAT-6, TB10.4 (p.p.) *Pre-challenge*: (+10 weeks) *Post-Challenge*: (+7, +26, +50 weeks)	BCG/H1:CAF01 and BCG induced a minor subset of memory 3^+^ T cells in lung pre-challenge For BCG/H1:CAF01, both an increased 3^+^ T cell response and decreased CFU in the lung relative to control mice were observed 26 weeks after infection. However, when comparing BCG/H1:CAF01 with BCG post-infection, BCG/H1:CAF01 was associated with an increased 3^+^ T cell response but not a statistically significant decrease in lung CFU	Lindenstrom et al. (38)

BCG (Pasteur) Ad85A	*Mouse strain*: Balb/c *Immunization*: BCG s.c. (+0)/Ad85A i.d. (+10 weeks); vs BCG s.c. (+0)/Ad85A i.n. (+10 weeks)	*Cell measured*: CD4^+^ 3^+^ cells *Cellular source*: spleen and lung *Antigen(s)*: Ag85A (p.p.) *Immunogenicity*: (+14 weeks)	Boosting BCG with Ad85A i.n. but not i.d. induced Ag85A-specific 3^+^ T cells in lung	Forbes et al. (39)

rESAT-6: CAF01	*Mouse strain*: CB6F1 *Immunization*: rESAT-6 s.c. in CAF01 (+0, +2, +4 weeks) *Challenge*: Mtb Erdman aerosol (+10 weeks) *Post-Challenge*: lung CFU (+4, +6, +10 weeks)	*Cell measured*: CD4^+^ 3^+^ cells *Cellular source*: lung *Antigen(s)*: ESAT-6_1–15_ peptide *Post-Challenge*: (+2, +6, +24 weeks)	For ESAT-6:CAF01, both an increased 3^+^ T cell response and decreased CFU in the lung relative to control mice were observed at all time points measured	Aagaard et al. (55)

H56:CAF01 H1:CAF01 BCG (SSI)	*Mouse strain*: CB6F1 *Immunization*: H56 s.c. in CAF01 (+0, +2, +4 weeks); H1 s.c. in CAF01 (+0, +2, +4 weeks) BCG (+0) *Challenge*: Mtb Erdman aerosol (+10 weeks) *Post-Challenge*: lung CFU (+6 weeks)	*Cell measured*: CD4^+^CD44^hi^ 3^+^ cells *Cellular source*: lung *Antigen(s)*: rAg85B *Post-Challenge*: (+6, +12, +24 weeks)	For H56:CAF01 relative to control, both an increased 3^+^ T cell response and decreased CFU in the lung were observed at all time points measured For H56:CAF01 relative to H1:CAF01, both an increased 3^+^ T cell response and decreased CFU in the lung were observed at +12 and +24 weeks For H56:CAF01 relative to BCG, both an increased 3^+^ T cell response and decreased CFU in the lung were observed at +24 weeks only	Aagaard et al. (56)

H56:CAF01	*Mouse strain*: CB6F1 *Immunization*: H56 s.c. in CAF01 (+0, +2, +4 weeks) *Challenge*: Mtb Erdman aerosol (+10 weeks) *Post-Challenge*: lung CFU (+42 days)	*Cell measured*: CD4^+^ 3^+^ cells *Cellular source*: lung *Antigen(s)*: rESAT-6 *Post-Challenge*: (+42 days)	For H56:CAF01 relative to control, both an increased 3^+^ T cell response and decreased CFU in the lung were observed at +42 days	Woodworth et al. (57)

BCG (SSI) VPM1002	*Mouse strain*: Balb/c *Immunization*: BCG s.c. (+0); VPM1002 (+0) *Challenge*: Mtb H37Rv aerosol *Post-Challenge*: lung CFU (+90 days)	*Cell measured*: CD4^+^ 3^+^ cells *Cellular source*: lung *Antigen(s)*: PPD *Post-Challenge*: (+7, +90 days)	VPM1002 vaccination prior to challenge resulted in greater frequencies of 3^+^ T cells in the lung as compared to BCG-immunized or control mice 7 days after challenge. Ninety days after challenge, frequencies of 3^+^ T cells in the lung were comparable in VMP1002-immunized, BCG-immunized and control mice. At this same time point, VMP1002-immunized mice controlled infection better than BCG-immunized mice	Desel et al. (58)

H4:CAF01 Ad-H4 BCG (SSI)	*Mouse strain*: CB6F1 *Immunization*: Ad-H4 s.c. (+0) vs H4 in CAF01 s.c. (+0, +2 weeks) vs H4 in CAF01 s.c. (+0)/Ad-H4 (+2 weeks) vs BCG s.c. (+0) *Challenge*: Mtb Erdman aerosol (+8 weeks) *Post-Challenge*: lung CFU (+6 weeks)	*Cell measured*: CD4^+^CD44^hi^ 3^+^ cells *Cellular source*: lung *Antigen(s)*: TB10.4; Ag85B (p.p.) *Post-Challenge*: (+2 weeks)	For H4:/Ad-H4 relative to control, both an increased 3^+^ T cell response (+2 weeks) and decreased CFU in the lung (+6 weeks) were observed	Elvang et al. (40)

BCG (SSI)	*Monkeys*: *Macaca mulatta* *Immunization*: BCG aerosol (+0)	*Cell measured*: CD4^+^ 3^+^ cells *Cellular source*: BAL *Antigen(s)*: PPD *Immunogenicity*: (+4, +8, +13 weeks)	BCG induced 3^+^ T cells as a major subset in BAL fluid 8 weeks after vaccination	White et al. (46)

AERAS-402	*Monkeys*: *M. mulatta* *Immunization*: AERAS-402 *via* aerosol (+1, +8, +15 days)	*Cell measured*: CD4^+^ 3^+^ cells *Cellular source*: BAL *Antigen(s)*: Ag85A/B (p.p.) *Immunogenicity*: (+18, +43 days)	AERAS-402 delivered *via* aerosol induced weak, detectable 3^+^ T cells in BAL 28 days after completing immunization	Hokey et al. (48)

*SSI, Staten Serum Institute; H1, Recombinant Ag85B/ESAT-6 fusion protein; CAF01, cationic liposomes formulated with synthetic mycobacterial cord factor; Ad85A, human adenoviral vector expressing Ag85A; rESAT-6, recombinant ESAT-6; H56, Recombinant Ag85B/ESAT-6/Rv2660c; VPM1002, Recombinant BCG which is urease C deficient and expressing membrane-perforating listerolysin (*L. monocytogenes*); H4, Recombinant Ag85B/TB10.4 fusion protein; Ad-H4, adenoviral vector expressing H4; AERAS-402, Adenoviral vector (Ad35) expressing Ag85A, Ag85B, and TB10.4; CB6F1, C57BL/6 X Balb/c; i.m., intramuscular; s.c., subcutaneous; i.d., intradermal; i.n., intranasal; CFU, colony forming units; 3^+^, IFN-γ^+^TNF-α^+^IL-2^+^; BAL, bronchoalveolar lavage; p.p., peptide pool; rAg85B, recombinant Ag85B; rESAT-6, recombinant ESAT-6; BCG, Bacille Calmette Guerin; TB, Tuberculosis; Mtb, *M. tuberculosis*; IA, immune assay*.

*Major subset = subset that constitutes >50% of the total cytokine-producing cells; minor subset = subset that constitutes <20% of the total cytokine-producing cells*.

Studies utilizing the mouse TB model have also investigated the effect of prior vaccine administration on the magnitude of the polyfunctional CD4^+^ T cell response in the lung following Mtb challenge (Table 3). Vaccination with recombinant ESAT-6 in CAF01 (rESAT-6:CAF01) (55) or Ag85B/ESAT-6/Rv2660c fusion protein in CAF01 (H56:CAF01) (56, 57), or VPM1002 (58), recombinant Ag85B/TB10.4 fusion protein in CAF01 (H4:CAF01), boosted with an Ad5 vector expressing recombinant H4 (Ad-H4) (40), or BCG boosted with Ag85B-ESAT-6 fusion protein in CAF01 (H1:CAF01) (38) all resulted in increased Mtb antigen-specific polyfunctional CD4^+^ T cell responses in lung relative to control mice 2 weeks (40, 55) up to 26 weeks (38) after Mtb challenge. In most of these studies both an increased polyfunctional CD4^+^ T cell response and vaccine-induced protection in the lung, as measured by decreased CFU in the lung relative to control mice, were observed at the same time point after infection. However, Desel et al. showed that 90 days after challenge, frequencies of polyfunctional T cells in the lung were comparable in VMP1002-immunized, BCG-immunized and control mice (58). Despite that, at this same time point, VMP1002-immunized mice controlled infection better than BCG-immunized mice and BCG-immunized mice controlled infection better than naïve mice. Also, Lindenstrom et al. showed that vaccination with BCG boosted with H1:CAF01 resulted in increased PPD-specific polyfunctional CD4^+^ T cell responses in lung relative to control mice and BCG-vaccinated mice, yet differences in lung CFU in BCG/H1:CAF01 vs BCG-vaccinated mice were not statistically significant (38). In another study, vaccination with recombinant Ag85B/ESAT-6/Rv2660c fusion protein (H56:CAF01) resulted in Ag85B-specific polyfunctional CD4^+^ T cells in the lungs, which were not detected in BCG-immunized or control mice, yet both BCG and H56-vaccinated mice demonstrated a similar reduction in lung CFU as compared to control mice at 6 and 12 weeks after challenge (56). In conclusion, in animal models, TB vaccines induce polyfunctional CD4^+^ T cells that are present in lung after immunization and in some, but not all studies, an increase in polyfunctional CD4^+^ T cells in the lung following Mtb challenge is temporally associated with vaccine-induced control of bacterial replication in the lung relative to control mice.

## Correlation of Polyfunctional T Cell Responses and Protection in the Mouse Model

Whether or not vaccine-induced polyfunctional CD4^+^ T cells represent a correlate of protective immunity from Mtb infection has been addressed in several mouse studies investigating the correlation between the magnitude of the polyfunctional CD4^+^ T cell response present before Mtb challenge and vaccine-induced control of bacterial replication, the most common measure of vaccine protection against TB in the murine model (Table 2). The strongest correlative evidence for polyfunctional CD4^+^ T cells as a correlate of protective immunity comes from studies in which a correlation between control of bacterial replication and the magnitude of the CD4^+^ T cell response was established from experiments including multiple distinct vaccine candidates that elicit a range of protective responses. Conversely, the weakest correlative evidence comes from studies in which a single vaccine candidate both induces a polyfunctional CD4^+^ T cell response and demonstrates protection compared to unimmunized control mice. For example, BCG (9, 41), H1:CAF01 (38, 41), and ID93:GLA-SE (54), all induced mycobacteria-specific polyfunctional CD4^+^ T cell responses at the time of Mtb challenge, which were associated with protection as compared to naïve or control mice, who lack these T cell responses. Somewhat better correlative evidence comes from studies comparing two or more vaccine candidates to one another. Comparing rBCG:XB to BCG, Yuan et al. showed that rBCG:XB elicited stronger HspX-specific polyfunctional T cell responses than BCG, which was associated with greater protection than was observed in BCG-vaccinated mice (44). Ballester et al. showed that recombinant Ag85B delivered in CpG (Ag85B:CpG) with polypropylene sulfide nanoparticles (NP) delivered i.n. induced more polyfunctional Ag85B-specific CD4^+^ T cells in spleen pre-challenge than did Ag85B:CpG without NP and this correlated with better protection in the lung (50). Also, comparing priming alone with prime/boost strategies, Elevang et al. demonstrated that recombinant Ag85B/TB10.4 fusion protein in CAF01 (H4:CAF01), boosted with an Ad5 vector expressing recombinant H4 (Ad-H4) induced more Ag85A- and TB10.4-specific polyfunctional memory CD4^+^ T cells, pre-challenge than did either recombinant H4 or Ad-H4 alone, which in turn correlated with better protection in lung after challenge (40). In addition, comparing different dosages of recombinant antigen, Aagaard et al. showed that H4:IC31 induced stronger Ag85B- and TB10.4-specific polyfunctional CD4^+^ T cells in PBMC pre-challenge at a lower dose (0.5 μg) vs a higher dose (5.0 μg), and this correlated with better protection in the lung at the lower dose (52). Finally, the strongest degree of correlation was demonstrated using several different vaccine regimens inducing various levels of protection (43). Derrick et al. showed that both the frequency of polyfunctional CD4^+^ T cells and the iMFI for TNF-α and IFN-γ at the time of Mtb challenge correlated with vaccine-induced protection in the lung and spleen over a 14-month period. For example, the magnitude of BCG-specific CD4^+^ polyfunctional T cell responses at the time of challenge 8 months after immunization with BCG or BCG mixed with recombinant E6-85, were greater than those induced by a DNA construct expressing recombinant E6-85 and correlated with greater protection afforded by either of these vaccine BCG regimens than the DNA construct. Moreover, using Pearson correlation analysis of iMFI values at all the challenge time points, these investigators showed that IFN-γ iMFI values were highly correlated with protection in the lung and spleen. Yet, of note, these investigators only reported measurements of the polyfunctional CD4^+^ T cell response, while other immune parameters that may also be mediators of protection, such as antibody responses and CD8^+^ T cell responses, were not evaluated for correlation with control of bacterial replication.

By contrast, two other studies show either equivocal evidence for or evidence against a correlation between pre-challenge polyfunctional CD4^+^ T cells and vaccine-induced protection. For example, boosting BCG with an Ad5 vector expressing Ag85A, induced Ag85A-specific polyfunctional CD4^+^ T cells pre-challenge in the lung only when delivered i.n. but not i.d. and this correlated with improved protection in the lung in i.n. vaccinated mice (39). However, i.d. rather than i.n. delivery induced polyfunctional CD4^+^ T cells in the spleen, such that only lung, not splenic polyfunctional CD4^+^ T cells correlated with the degree of vaccine-induced protection. In another study, both BCG and recombinant BCG (VPM1002) required boosting with MVA85A to elicit PPD- and Ag85A-specific polyfunctional CD4^+^ T cells, with BCG boosted with MVA85A eliciting greater T cell responses than VPM1002 boosted with MVA85A (42). Yet VPM1002 provided better protection in lung compared to BCG and the MVA85A boost, which induced high levels of polyfunctional CD4^+^ T cells, did not augment protection obtained with either BCG or VPM1002. Differences in these two studies as compared to numerous studies that support a correlation between polyfunctional CD4^+^ T cells are difficult to resolve, but may simply reflect differences in the vaccination protocol, or how polyfunctional T cells or protection was measured. In addition, two studies showed a stronger correlation between dual IL-2 and TNF-α producing CD4^+^ T cells and vaccine-induced protection than with triple IL-2, TNF-α, and IFN-γ-producing CD4^+^ T cells (38, 41). Finally, Yuan et al. showed a stronger correlation between the magnitude of dual TNF-α and IFN-γ-producing CD4^+^ T cells and vaccine-induced protection than with triple IL-2, TNF-α, and IFN-γ-producing CD4^+^ T cells (44). In summary, studies of the role of polyfunctional CD4^+^ T cells in mediating vaccine-induced protection in the mouse TB vaccine model provide evidence that these T cell responses represent at best an imperfect correlate of protection. In addition, these data can neither confirm nor refute CD4^+^ polyfunctional T cells as a mechanistic correlate of protection—i.e., a causal relationship between the vaccine-induced polyfunctional CD4^+^ T cell responses and control of bacterial replication following challenge is uncertain and cannot be ruled in or ruled out based upon these studies.

## TB Vaccines Induce Polyfunctional T Cells in Humans

Bacille Calmette Guerin and novel TB vaccine candidates utilizing various antigens and vaccine platforms induce polyfunctional CD4^+^ T cells in infants, older children and adults. These T cell populations include memory T cell populations that persist for months to years after vaccination.

### BCG and Other Live Mycobacterial Vaccines Elicit Polyfunctional T Cells

Several studies have demonstrated that BCG induces CD4^+^ polyfunctional T cells in infants (Table 4). BCG induces mycobacteria-specific polyfunctional CD4^+^ T cells, in healthy infants from South Africa (59–62), Uganda (63), Australia (64, 65), and the UK (66). These T cell responses represented the predominant subset among cytokine-producing T cells (59, 60, 66) peaked between 6 and 10 weeks of age and persisted for at least 1 year (60, 66). Comparison to unimmunized infants demonstrated that the polyfunctional T cell response was elicited by BCG and not by exposure to environmental mycobacteria (60, 66). Studies of Australian infants (65), South African infants (60), and Ugandan infants (63) compared BCG immunization at birth to delayed immunization at 6–10 weeks. These studies in general demonstrated no differences in polyfunctional CD4^+^ T cell responses during the first few weeks following immunization. However, South African infants who received delayed BCG had higher polyfunctional CD4^+^ T cell responses at 1 year of age than did those receiving BCG at birth (60). Also, Ritz et al. showed that BCG (Denmark, SSI) and BCG (Japan, Tokyo-172) induced higher polyfunctional CD4^+^ T cell responses than did BCG (Russian, SL-222), which correlated with more severe local reactions to the vaccine (64). BCG at birth also induced polyfunctional CD4^+^ T cell responses in HIV-infected infants, though at decreased numbers relative to HIV-exposed/uninfected infants and HIV-unexposed infants (61). Finally, Loxton et al. compared the immunogenicity of BCG to recombinant BCG, VPM1002, in healthy South African newborns and demonstrated comparable frequencies of polyfunctional CD4^+^ T cell responses 6 weeks, 18 weeks and 6 months after vaccination (67). Thus, ample evidence demonstrates that BCG induces polyfunctional CD4^+^ T cells in human infants.

**Table 4:** TB vaccines induce polyfunctional CD4^+^ T cells in human infants.

Vaccine	Study design	IA	Conclusions	Reference
BCG (SSI)	*Cohort(s)*: healthy South African infants born at term; HIV-uninfected/unexposed, TB uninfected/unexposed *Immunization*: BCG i.d. (+0) *Randomized*: birth (*n* = 25) vs 10 weeks (*n* = 21)	*Cell measured*: CD4^+^ 3^+^ cells *Cellular Source*: whole blood *Antigen(s)*: BCG *Immunogenicity*: (+10, +20, +50 weeks)	3^+^ T cells were readily detected in both cohorts, which peaked 10 weeks after vaccination. No 3^+^ T cells were detected pre-vaccination in the 10-weeks delayed cohort. 3^+^ T cells persisted at 1 year and the magnitude was greater in the BCG delayed than in the BCG at birth cohort	Kagina et al. (60)

BCG (Japanese)	*Cohort(s)*: 2-year follow-up of healthy South African infants born at term HIV-uninfected/unexposed TB (Cx^+^; *n* = 29) Healthy Mtb exposed (*n* = 55) Healthy Mtb unexposed (*n* = 55) *Immunization*: BCG (+0)	*Cell measured*: CD4^+^ 3^+^ cells *Cellular Source*: whole blood *Antigen(s)*: BCG *Immunogenicity*: (+10 weeks)	3^+^ T cells were equivalent in the TB, TB exposed, and TB unexposed cohorts. There was no correlation between 3^+^ T cells at 10 weeks and development of TB within 2 years	Kagina et al. (62)

BCG (SSI)	*Cohort(s)*: South African infants born at term HIV-infected (ART naïve, *n* = 20) HIV-exposed (*n* = 25) HIV-unexposed (*n* = 25) HIV was ART naïve *Immunization*: BCG i.d. (+0)	*Cell measured*: CD4^+^ 3^+^ cells *Cellular Source*: whole blood *Antigen(s)*: BCG *Immunogenicity*: (+3, +6, +9, +12 months)	3^+^ T cells were equivalent in the HIV-exposed and HIV-unexposed cohorts and decreased in the HIV-infected, ART naïve cohort. 3^+^ T cells peaked at 3 months in all cohorts	Mansoor et al. (61)

BCG (SSI)	*Cohort(s)*: healthy South African infants born at term; HIV-uninfected/unexposed, TB uninfected/unexposed (*n* = 29) *Immunization*: BCG i.d. (+0)	*Cell measured*: CD4^+^ 3^+^ cells *Cellular Source*: whole blood *Antigen(s)*: BCG *Immunogenicity*: (+10 weeks)	3^+^ T cells were readily detected at 10 weeks after immunization	Soares et al. (59)

BCG	*Cohort(s)*: healthy BCG-immunized Ugandan infants, 9 months old; HIV-uninfected/unexposed, Mtb unexposed *Immunization*: BCG at birth (*n* = 50) BCG at 6 weeks of age: (*n* = 42)	*Cell measured*: CD4^+^ 3^+^ cells *Cellular Source*: whole blood *Antigen(s)*: BCG *Immunogenicity*: once at 9 months of age	3^+^ T cells were readily detected in both cohorts and were of comparable magnitude in infants immunized at birth and at 6 weeks of age	Lutwama et al. (63)

BCG Danish (SSI) Japan (Tokyo-172) Russia (SL-222)	*Cohort(s)*: healthy Australian infants born at term; HIV-uninfected/unexposed, TB uninfected/unexposed *Immunization*: BCG i.d. (+0) Denmark (*n* = 54) Japan (*n* = 54) Russia (*n* = 57)	*Cell measured*: CD4^+^ 3^+^ cells *Cellular Source*: whole blood *Antigen(s)*: BCG; PPD; heat-killed Mtb (H37Rv) *Immunogenicity*: (+10 weeks)	3^+^ T cells were equivalent in infants immunized with the Danish and Japanese BCG strains. BCG- and PPD-specific 3^+^ T cells, as well as the local reaction sizes, were greater in infants immunized with the Danish and Japanese BCG strains, than in those immunized with the Russian BCG strain	Ritz et al. (64)

BCG (SSI)	*Cohort(s)*: healthy Australian infants born at term; HIV-uninfected/unexposed *Immunization*: BCG i.d. (+0) Birth (*n* = 54) 2 months (*n* = 44)	*Cell measured*: CD4^+^ 3^+^ cells *Cellular Source*: whole blood *Antigen(s)*: BCG; PPD; heat-killed Mtb (H37Rv) *Immunogenicity*: (+10 weeks)	3^+^ T cells were a minor subset and equivalent in infants immunized at birth and at 2 months of age	Ritz et al. (65)

BCG (SSI)	*Cohort(s)*: healthy term infants born in the UK; HIV-uninfected/unexposed *Immunization*: BCG i.d. at 5.6 (4.3–8 weeks; *n* = 24) BCG naïve (*n* = 15)	*Cell measured*: CD4^+^ 3^+^ cells *Cellular Source*: whole blood *Antigen(s)*: PPD *Immunogenicity*: (+4 months and +1 year of age)	3^+^ T cells were a major subset at 4 months and 1 year of age. No 3^+^ T cells were detected in BCG naïve infants	Smith et al. (66)

BCG (SSI) VPM1002	*Cohort(s)*: open-label, randomized Phase II study of healthy South African infants born at term; HIV-uninfected/unexposed *Immunization*: BCG i.d. (+0; *n* = 12) VPM1002 i.d. (+0; *n* = 36)	*Cell measured*: CD4^+^ 3^+^ cells *Cellular Source*: whole blood *Antigen(s)*: PPD, BCG *Immunogenicity*: (+6, +18 weeks, +6 months)	3^+^ T cells were equivalent in infants immunized with BCG or VPM1002, 6 weeks, 18 weeks, and 6 months after vaccination	Loxton et al. (67)

BCG AERAS-402	*Cohort(s)*: double-blinded, randomized placebo-controlled trial in South Africa, Kenya, and Mozambique Healthy BCG-immunized infants, 16–26 weeks-old; HIV-uninfected; Mtb-uninfected *Immunization*: AERAS-402 i.m. (+0, +28, +280 days; *n* = 60) Placebo (vaccine buffer) i.m. (+0, +28, +280 days; *n* = 55)	*Cell measured*: CD4^+^ 3^+^ cells *Cellular Source*: PBMC *Antigen(s)*: Ag85A; Ag85B; TB10.4 (p.p.) *Immunogenicity*: [+308, study end (+448–664 days)]	3^+^ T cells were detected 308 days after the first immunization and at the end of the study (448–664 days). These responses were lower than had been observed in BCG-immunized adults in a prior study	Tameris et al. (68)

BCG MVA85A	*Cohort(s)*: open-label Phase 2a trial of healthy BCG-immunized South African infants, 5–12 months old; HIV-uninfected, Mtb-uninfected *Immunization*: MVA85A i.d. (+0; *n* = 18) Prevnar i.m. (*n* = 12)	*Cell measured*: CD4^+^ 3^+^ cells; GM-CSF; IL-17 *Cellular Source*: whole blood *Antigen(s)*: Ag85A (p.p.) *Immunogenicity*: [+0, +28, +168 days; and 3.3 (3.2–3.5 years)]	MVA85A boost to BCG induced a major subset of 3^+^ T cells, the majority of which co-expressed GM-CSF, and a minority of which also co-expressed IL-17. These responses peaked at 28 days and persisted over 3 years following immunization. 3^+^ T cells at 3 years displayed predominantly an effector memory phenotype (CD45RA^neg^CCR7^neg^)	Scriba et al. (69) and Tameris et al. (70)

BCG (SSI) MVA85A	*Cohort(s)*: double-blind Phase 2b trial in healthy BCG-immunized South African infants 4–6 months old; HIV-uninfected, Mtb-uninfected/unexposed *Immunization*: MVA85A i.d. (+0; *n* = 17) Candin i.d. (+0; *n* = 19)	*Cell measured*: CD4^+^ 3^+^ cells *Cellular Source*: whole blood *Antigen(s)*: Ag85A (p.p.) *Immunogenicity*: (+0, +28 days)	MVA85A boost to BCG elicited 3^+^ T cells 28 days after immunization, which were not present prior to vaccination or in placebo recipients. No significant efficacy against Mtb infection or TB disease as compared to BCG alone was observed. These 3^+^ T cell responses were lower than had been observed in BCG-immunized adults in a prior study	Tameris et al. (3)

*SSI, Staten Serum Institute; VPM1002, recombinant BCG which is urease C deficient and expressing membrane-perforating listerolysin (*L. monocytogenes*); AERAS-402, adenoviral vector (Ad35) expressing Ag85A, Ag85B, and TB10.4; MVA85A, modified vaccinia Ankara expressing Ag85A. ART, anti-retroviral therapy; Prevnar, pneumococcal 7-valent conjugate vaccine (Wyeth); Candin, *Candida* skin test antigen; i.m., intramuscular; i.d., intradermal; 3^+^, IFN-γ^+^TNF-α^+^IL-2^+^; PBMC, peripheral blood mononuclear cells; p.p., peptide pool; BCG, Bacille Calmette Guerin; TB, tuberculosis; Mtb, *M. tuberculosis*; IA, immune assay*.

*Minor subset = subset that constitutes <20% of the total cytokine-producing cells; major subset = subset that constitutes >50% of the total cytokine-producing cells*.

Fewer studies have evaluated polyfunctional CD4^+^ T cells in BCG-immunized adults (Table 5). Two small studies of adults with a remote history of BCG immunization demonstrated mycobacteria-specific polyfunctional CD4^+^ T cell responses in these individuals, which represented the predominant subset of cytokine-producing cells (9, 71) and displayed a memory phenotype (71). In a small study of BCG naïve, Mtb-uninfected adults immunized with BCG, BCG-specific polyfunctional CD4^+^ T cell responses were variably detected and when present, peaked 8 weeks following vaccination, demonstrated an effector phenotype and correlated with local inflammation at the vaccination site (72). These responses waned by 1 year after vaccination. Consistent with these results, no polyfunctional CD4^+^ T cell responses were observed in British adolescents 1 year after BCG vaccination (73). Finally, Spertini et al. compared the immunogenicity of BCG with MTBVAC, a live attenuated strain of Mtb, in a randomized double-blind Phase I trial of BCG naïve, Mtb-uninfected Swiss adults (74). Both BCG and MTBVAC induced MTBVAC- and BCG-specific polyfunctional CD4^+^ T cell responses that persisted up to 210 days after vaccination. Therefore, although the evidence is less abundant than for infants, live mycobacterial vaccines also induce polyfunctional CD4^+^ T cell responses in adults.

**Table 5:** TB vaccines induce polyfunctional CD4^+^ T cells in human children, adolescents, and adults.

Vaccine	Study design	IA	Conclusions	Reference
BCG	*Cohort(s)*: BCG-immunized adults from the United States; Mtb-uninfected (RD-1 ELISPOTneg), no history of TB (*n* = 20) *Immunization*: BCG (remote)	*Cell measured*: CD4^+^ 3^+^ cells *Cellular Source*: PBMC *Antigen(s)*: Mtb cell wall *Immunogenicity*: once (unknown timing)	3^+^ T cells were detected as a major subset in adults with a remote history of BCG immunization	Adekambi et al. (71)

BCG	*Cohort(s)*: BCG-immunized adults (*n* = 4) *Immunization*: BCG (remote)	*Cell measured*: CD4^+^ 3^+^ cells *Cellular Source*: PBMC *Antigen(s)*: PPD *Immunogenicity*: once (unknown timing)	3^+^ T cells were detected as a major subset in adults with a remote history of BCG immunization	Darrah et al. (9)

BCG (SSI)	*Cohort(s)*: BCG naïve adults from the Netherlands; Mtb-uninfected (TSTneg, QFTneg, *n* = 12) *Immunization*: BCG i.d. (+0)	*Cell measured*: CD4^+^ 3^+^ cells *Cellular Source*: PBMC *Antigen(s)*: BCG *Immunogenicity*: (+0, +4, +8, +12, +52 weeks)	BCG induced 3^+^ T cells in some individuals with a greater local skin reaction. When observed, 3^+^ T cells responses peaked 8 weeks after immunization	Boer et al. (72)

BCG (SSI)	*Cohort(s)*: BCG naïve adolescents from UK (*n* = 8) *Immunization*: BCG (+0)	*Cell measured*: CD4^+^ 3^+^ cells *Cellular Source*: PBMC *Antigen(s)*: PPD *Immunogenicity*: (+12 months)	3^+^ T cells were not detectable in adolescents 1 year after BCG vaccination	Smith et al. (73)

MTBVAC BCG (SSI)	*Cohort(s)*: randomized double-blind Phase I trial of BCG naive adults from Switzerland; BCG naïve, HIV-uninfected, Mtb-uninfected (RD-1 ELISPOTneg) *Immunization*: MTBVAC i.d. (5 × 10^3^, 5 × 10^4^, 5 × 10^5^ CFU; +0, *n* = 9, each dose) BCG i.d. (+0, *n* = 9)	*Cell measured*: CD4^+^ 3^+^ cells *Cellular Source*: whole blood *Antigen(s)*: MTBVAC; BCG *Immunogenicity*: (+0, +28, +90, +210 days)	BCG and MTBVAC induced 3^+^ T cells to both BCG and MTBVAC stimulations. MTBVAC at the highest dose (5 × 10^5^ CFU) induced 3^+^ T cells, which were still detectable 210 days after immunization	Spertini et al. (74)

H4:IC31	*Cohort(s)*: double-blind, Phase I trial of South African adults; BCG-immunized (remote), HIV-uninfected, Mtb-uninfected (QFTneg), no TB *Immunization*: H4 in IC31 i.m. (5, 15, 50, 150 μg; +0, +56 days; *n* = 8, each dose) Saline i.m. (+0, +56 days; *n* = 8)	*Cell measured*: CD4^+^ 3^+^ cells *Cellular Source*: PBMC *Antigen(s)*: Ag85B; TB10.4 (p.p.) *Immunogenicity*: (+0, +14, +28, +56, +70, +84, +182 days)	H4:IC31 induced 3^+^ T cells in Mtb-uninfected, BCG-immunized adults. While all doses elicited 3^+^ cells, lower doses induced greater frequency 3^+^ T cells than did the highest dose (150 μg) and the highest magnitude response was 84 days after immunization (15 μg)	Geldenhuys et al. (75)

H56:IC31	*Cohort(s)*: open-label Phase I trial of South African adults; BCG-immunized (remote, assumed), HIV-uninfected, no TB disease, Mtb infections defined with QFT *Immunization*: rH56 in IC31 i.m. (+0, +56, +112 days) Mtb-uninfected (50 μg, *n* = 8) Mtb-infected (15 μg, *n* = 8) Mtb-infected (50 μg, *n* = 8)	*Cell measured*: CD4^+^ 3^+^ cells, CD45RA, CCR7 *Cellular Source*: whole blood *Antigen(s)*: H56 *Immunogenicity*: (+0, +70, +210 days)	H56:IC31 induced 3^+^ T cells in both Mtb-infected and Mtb-uninfected BCG-immunized adults. 3^+^ T cell responses were greater in Mtb-infected than in Mtb-uninfected individuals, comprised a predominant subset at +70 days and persisted at least to +210 days. At +210 days, 3^+^ T cells displayed a central memory (CD45RA-CCR7^+^) or effector memory (CD45RA-CCR7-) phenotype. Among Mtb-infected individuals, the low dose (15 μg) elicited greater frequencies of 3^+^ T cells than the high dose (50 μg)	Luabeya et al. (76)

H1:IC31	*Cohort(s)*: observer-blinded Phase II trial of South African adolescents (12–18 years old); BCG-immunized at birth, HIV-uninfected, no TB disease, comparison of Mtb-infected (QFTpos; *n* = 25) to Mtb-infected (QFTneg; *n* = 35) individuals *Immunization*: H1 in IC31 i.m. (15 μg; +0, +56 days)	*Cell measured*: CD4^+^ 3^+^ cells *Cellular Source*: whole blood *Antigen(s)*: Ag85B; ESAT-6 (p.p.) *Immunogenicity*: (+0, +14, +56, +70, +224 days)	H1:IC31 induced 3^+^ T cell responses over baseline in both Mtb-infected and Mtb-uninfected adolescents, which comprised a predominant subset in both cohorts, and persisted at least to +70 days in Mtb-infected and to +224 days in Mtb-uninfected individuals	Mearns et al. (77)

H1:IC31	*Cohort(s)*: randomized double-blind Phase II trial of HIV-infected adults from Tanzania; CD4 counts >350, ARV naïve, no TB disease *Immunization*: H1 in IC31 i.m. (+0, +56 days; *n* = 20) Buffer i.m. (+0, +56 days; *n* = 4)	*Cell measured*: CD4^+^ 3^+^ cells *Cellular Source*: whole blood *Antigen(s)*: H1 *Immunogenicity*: (+0, +14, +56, +70, +182 days)	H1:IC31 induces a predominant subset of 3^+^ T cells in HIV-infected adults, which peak 70 days and persist at least 182 days after initiation of immunization	Reither et al. (78)

Mtb72F: AS02_A_	*Cohort(s)*: randomized observer blind Phase I/II trial of adults from Switzerland; HIV-uninfected, compared BCG-immunized (remote, *n* = 20) to Mtb-infected (TST^+^, *n* = 18; included subset with history of TB disease, *n* = 5) *Immunization*: Mtb72F in AS02_A_ i.m. (+0, +1, +3 months) AS02_A_ i.m. (+0, +1, +3 months)	*Cell measured*: CD4^+^ 3^+^ cells, CD40L *Cellular Source*: PBMC *Antigen(s)*: Mtb32A; Mtb39A (p.p.) *Immunogenicity*: (+0, +60, +90, +240 days)	In both Mtb-infected and BCG-immunized adults, 3^+^ T cells were detected before immunization. Also in both cohorts, Mtb72F:AS02_A_, but not AS02_A_ alone, resulted in increased 3^+^ T cells after the second vaccination, which were not further boosted by the third vaccination, and persisted at least to +240 days. A predominant subset in both cohorts also expressed CD40L	Spertini et al. (79)

M72:AS01_B_ M72:AS02_A_	*Cohort(s)*: randomized observer blind Phase I/II trial of adults from Belgium; HIV-uninfected, Mtb-uninfected (TSTneg) *Immunization*: M72 in AS01_B_ i.m. (+0, +1 month; *n* = 40) M72 in AS02_A_ i.m. (+0, +1 month; *n* = 40)	*Cell measured*: CD4^+^ 3^+^ cells, CD40L *Cellular Source*: PBMC *Antigen(s)*: M72 (p.p.) *Immunogenicity*: (+0, +1, +2, +12, +24, +36 months)	M72:AS01_B_ and M72:AS02_A_ induced 3^+^ T cells first detected 1 month after the second immunization (+2 months), which persisted to least 36 months. 3^+^ T cell responses were greater in M72:AS01_B_ than in M72:AS02_A_ immunized individuals. The majority of the 3^+^ T-cell response co-expressed CD40L	Leroux-Roels et al. (80)

M72: AS01_E_	*Cohort(s)*: open-label, Phase II trial of South African adults; HIV-uninfected; BCG-immunized (remote), Mtb-infected (TSTpos, 67%); no TB disease (*n* = 45) *Immunization*: M72 in AS01_E_ i.m. (+0, +30 days)	*Cell measured*: CD4^+^ 3^+^ cells *Cellular Source*: whole blood *Antigen(s)*: M72 *Immunogenicity*: (+0, +7, +30, +37, +60, +210 days)	M72:AS01_E_ induced 3^+^ T cell responses over baseline, which comprised a predominant subset, and persisted at least to +210 days	Day et al. (81)

M72:AS01_E_	*Cohorts*: double-blind, randomized Phase II trial of South African adolescents; HIV-uninfected, BCG-immunized (at birth); Mtb-infected (QFTpos; 53%); no TB disease *Immunization*: M72 in AS01_E_ i.m. (+0, +30 days; *n* = 40) Saline i.m. (+0, +30 days; *n* = 20)	*Cell measured*: CD4^+^ 3^+^ cells, CD40L *Cellular Source*: whole blood and PBMC *Antigen(s)*: M72 (p.p.); M72 *Immunogenicity*: (+0, +7, +30, +37, +60, +120 days)	M72:AS01_E_ induced 3^+^ T cell responses over baseline, which comprised a predominant subset, and persisted at least to +210 days. 3^+^ T cells co-expressed CD40L	Penn-Nicholson et al. (82)

M72:AS01_E_	*Cohort(s)*: randomized, observer blind, Phase II trial of adults from India; no history of TB disease, comparison of HIV-infected on stable ART (*n* = 80) to ART naïve HIV-infected (*n* = 80) to HIV-uninfected (*n* = 80) *Immunization*: M72 in AS01_E_ i.m. (+0, +1 month; *n* = 40, each cohort) Saline i.m. (+0, +1 month; *n* = 40 each cohort)	*Cell measured*: CD4^+^ 3^+^ cells, CD40L *Cellular Source*: PBMC *Antigen(s)*: M72 (p.p.) *Immunogenicity*: (+0, +7, +30, +37, +60 days, +7, +13 months)	In all cohorts, 3^+^ T cells were detected before immunization. Also in the cohorts receiving, M72, but not those receiving placebo, M72 resulted in increased 3^+^ T cells, which peaked 37 days after immunization and persisted at least 13 months. A predominant subset in both cohorts also expressed CD40L	Kumarasamy et al. (83)

M72: AS01_E_	*Cohort(s)*: randomized, observer blind, Phase I/II trial of HIV-infected adults from Switzerland on ART; No history of TB disease, CD4 >200, BCG-immunized (73%), Mtb-infected (QFT^+^, 3%) *Immunization*: M72 in AS01_E_ i.m. (+0, +1 month; *n* = 22) AS01_E_ i.m. (+0, +1 month; *n* = 8) Saline i.m. (+0, +1 month; *n* = 7)	*Cell measured*: CD4^+^ 3^+^ cells, CD40L *Cellular Source*: PBMC *Antigen(s)*: M72 (p.p.) *Immunogenicity*: (+0, +30, +60, +201 days)	M72:AS01_E_ induced 3^+^ T cells in HIV-infected adults that peaked at +60 days and persisted at +210 days. All 3^+^ T cells co-expressed CD40L	Thacher et al. (84)

AERAS-402	*Cohort(s)*: randomized, double-blind, Phase I trial of BCG-immunized adults from South Africa; HIV-uninfected, Mtb-uninfected (QFTneg and TSTneg) *Immunization*: AERAS-402 i.m. (3 × 10^8^, 1.3 × 10^9^, 3 × 10^10^ VP; +0, *n* = 7, each dose) AERAS-402 i.m.(3 × 10^10^ VP; +0, +56; *n* = 7) Diluent i.m. (+0, ±56, *n* = 12)	*Cell measured*: CD4^+^ 3^+^ cells *Cellular Source*: PBMC and whole blood *Antigen(s)*: Ag85A/B, TB10.4 (p.p.) *Immunogenicity*: (0, +7, +28, +84, +182 days)	In BCG-immunized, Mtb-uninfected adults, AERAS-402 induced 3^+^ T cells to all vaccine components that were the predominant subset at +28 days, which persisted at +84 days, and were not detected at +182 days. No differences in immunogenicity were noted in the cohorts that received one dose compared to two doses	Abel et al. (85)

AERAS-402	*Cohort(s)*: HIV-infected, BCG-immunized South African adults; CD4 >350, no current ARV, Mtb-infected (QFT^+^, 50%) *Immunization*: AERAS-402 i.m. (+0, +1 month; *n* = 13) Buffer i.m. (+0, +1 month; *n* = 13)	*Cell measured*: CD4^+^ 3^+^ cells *Cellular Source*: PBMC *Antigen(s)*: Ag85A, Ag85B, TB10.4 (p.p.) *Immunogenicity*: (0, +7, +14, +28, +35, +42, +56, +84, +182 days)	In HIV-infected, BCG-immunized adults, AERAS-402 induced 3^+^ T cells to Ag85A/B, but not to TB10.4, that were the predominant subset and which peaked 2 weeks after the second immunization. 3^+^ T cell responses were not different in Mtb-infected as compared to Mtb-uninfected individuals	Churchyard et al. (86)

AdHu5Ag85A	*Cohort(s)*: Phase I trial of adults from Canada: HIV-uninfected; Mtb-uninfected (QFTneg), comparison of BCG-immunized (*n* = 12) to BCG naïve (*n* = 12) individuals *Immunization*:AdHu5Ag85A i.m. (+0)	*Cell measured*: CD4^+^ 3^+^ cells *Cellular Source*: PBMC *Antigen(s)*: Ag85A (p.p.); Mtb CF *Immunogenicity*: (+0, +2, +4, +8, +24 weeks)	AdHu5Ag85A induced 3^+^ T cells in both BCG-immunized and BCG naïve adults, which peaked 2–4 weeks after vaccination and at some time points represented a predominant subset. 3^+^ T cell responses where greater in BCG-immunized than BCG naïve adults	Smaill et al. (87)

MVA85A	*Cohort(s)*: open-label Phase I trial of adults from South Africa; HIV-uninfected, Mtb-uninfected (TSTneg), no TB disease, BCG-immunized (50%) *Immunization*: MVA85A i.d. (+0; *n* = 24)	*Cell measured*: CD4^+^ 3^+^ cells *Cellular Source*: whole blood *Antigen(s)*: BCG, Ag85A (p.p.) *Immunogenicity*: [−14, +7, +168 days, +5.7 (5.3–6.1) years]	In Mtb-uninfected adults, 3^+^ T cells were detected before immunization. Then MVA85A boosted 3^+^ T cells were a predominant subset and persisted for 5–6 years after immunization	Hawkridge et al. (88) and Tameris et al. (70)

MVA85A	*Cohort(s)*: randomized Phase I trial of BCG-immunized adults from the UK; HIV-uninfected, Mtb-uninfected (RD-1 ELISPOTneg) *Immunization*: MVA85A i.m. +0 (*n* = 12) MVA85 i.d. +0 (*n* = 12)	*Cell measured*: CD4^+^ 3^+^ cells *Cellular Source*: PBMC *Antigen(s)*: PPD; Ag85A (p.p.) *Immunogenicity*: (+0, +1, +24 weeks)	In BCG-immunized adults, MVA85A, delivered i.m. or i.d. induced 3^+^ T cells as a predominant subset equivalently and responses persisted for 24 weeks	Meyer et al. (89)

MVA85A	*Cohort(s)*: BCG-immunized adults from the UK; HIV-uninfected, Mtb-uninfected (TSTneg; *n* = 6) *Immunization*: MVA85A i.d. (+0)	*Cell measured*: CD4^+^ 3^+^ cells, MIP-1β, CD45RA/RO, CD27, CD57 *Cellular Source*: PBMC *Antigen(s)*: Ag85A (p.p.) *Immunogenicity*: (+1, 2, 8, 24 weeks)	MVA85A induced 3^+^ T cells in BCG-immunized adults, as a predominant subset that persisted at least 24 weeks. Predominant 3^+^ T cells subsets co-expressed MIP-1β. 3^+^ T cells demonstrated a phenotype consistent with immediate maturity (CD45ROneg/CD27neg/intermediate/CD57neg)	Beveridge et al. (90)

MVA85A	*Cohort(s)*: open-label Phase IIa trial of South African adults; Mtb infection defined by TST and RD-1 ELISPOT, compared HIV-uninfected, Mtb-infected (*n* = 12); HIV-infected, Mtb-uninfected (*n* = 12); HIV-infected, Mtb-infected (*n* = 12); HIV-infected on ARV (*n* = 12) *Immunization*: MVA85A i.d. (+0)	*Cell measured*: CD4^+^ 3^+^ cells *Cellular Source*: whole blood *Antigen(s)*: Ag85A (p.p.) *Immunogenicity*: (−7 to 14, +7, +28, +84, +364 days, 3–5 years)	3^+^ T cells were present prior to immunization in the Mtb-infected, but not the Mtb-uninfected cohorts. MVA85A induced 3^+^ T cells in all cohorts, as a predominant subset, which persisted 3–5 years, except for in the HIV-infected, Mtb-uninfected cohort	Scriba et al. (91) and Tameris et al. (70)

MVA85A	*Cohort(s)*: open-label Phase I trial of Mtb-infected adults from the UK; HIV-uninfected, Mtb infection defined with TST and RD-1 ELISPOT, no TB disease (*n* = 12) *Immunization*: MVA85A i.d. (+0)	*Cell measured*: CD4^+^ 3^+^ cells *Cellular Source*: PBMC *Antigen(s)*: Ag85A (p.p.) *Immunogenicity*: (+0, +1, +4, +24 weeks)	3^+^ T cells were present prior to immunization. MVA85A induced 3^+^ T cells as a major subset, which persisted at least 24 weeks	Sander et al. (92)

MVA85A	*Cohort(s)*: open label Phase I/IIa trial of South African, children age 4.3 (1.4–7.7) years (*n* = 24) and adolescents age (12–14 years, *n* = 12); HIV-uninfected, Mtb-uninfected; BCG-immunized at birth; no TB disease *Immunization*: MVA85A i.d. (+0)	*Cell measured*: CD4^+^ 3^+^ cells, IL-17, GM-CSF, CD45RA, CCR7 *Cellular Source*: whole blood *Antigen(s)*: BCG; Ag85A (p.p.); rAg85A *Immunogenicity*: adolescents: (+7, +28, +168 days, +4.6 [4.4–4.8] years) Children: (+7, +84, +168 days, +3.7 [3.7–3.9] years)	MVA85A induced 3^+^ T cells in both children and adolescents, as a predominant subset. 3^+^ T cells were greater in adolescents than children, peaked at +28 and +84 days, in adolescents and children, respectively, and persisted 3–5 years in both cohorts. In children, a major subset of 3^+^ T cells co-expressed IL-17 and GM-CSF. In adolescents, 3^+^ T cells co-expressed IL-17 and displayed an effector memory (CD45RAneg/CCR7neg) phenotype	Scriba et al. (69) and Tameris et al. (70)

*SSI, Staten Serum Institute; MTBVAC, a live attenuated strain of Mtb; H4, recombinant Ag85B/TB10.4 fusion protein; IC31, cationic peptide and oligodeoxynucleotide (ODN1); H56, recombinant Ag85B/ESAT-6/Rv2660c fusion protein; H1, recombinant Ag85B/ESAT-6 fusion protein; Mtb72F, recombinant Mtb32A/Mtb39A fusion protein; AS02_A_, MPL and QS21 in an oil-in-water emulsion; M72, Mtb72F with a point mutation in Mtb32A to improve stability; AS01_B_ and AS01_E_, 3-*O*-desacyl-4′-monophosphoryl lipid A (MPL) and *Quillaja saponaria* fraction 1 (QS21), combined with liposomes; AERAS-402, Adenoviral vector (Ad35) expressing Ag85A, Ag85B, and TB10.4; AdHu5Ag85A, human adenoviral vector (Ad5) expressing Ag85A; MVA85A, Modified Vaccinia Ankara expressing Ag85A; RD-1 ELISPOT, ELISPOT assay to detect PBMC secreting IFN-γ in response to RD-1 antigens, ESAT-6 and CFP-10 as evidence of Mtb infection; TST, tuberculin skin test; QFT, QuantiFERON assay including QuantiFERON Gold and QuantiFERON Gold in-tube; ARV, anti-retroviral therapy; i.m., intramuscular; i.d., intradermal; CFU, Colony Forming Units; VP, viral particles; 3^+^, IFN-γ^+^TNF-α^+^IL-2^+^; PBMC, peripheral blood mononuclear cells; p.p., peptide pools; Mtb CF, Mtb culture filtrate; rAg85A, recombinant Ag85A; BCG, Bacille Calmette Guerin; TB, tuberculosis; Mtb, *M. tuberculosis*; IA, immune assay*.

*Major subset = subset that constitutes >50% of the total cytokine-producing cells; predominant subset = subset that constitutes 20–50% of the total cytokine-producing cells*.

### Recombinant Protein Subunit Vaccines Elicit Polyfunctional T Cells

Several recombinant antigen TB vaccines have been studied in adults for their capacity to induce polyfunctional CD4^+^ T cell responses (Table 5). Recombinant Ag85B/TB10.4 fusion protein formulated with IC31 adjuvant (H4:IC31), induced H4-specific polyfunctional CD4^+^ T cells in previously BCG-vaccinated, Mtb-uninfected South African adults (75). While all doses induced these T cell responses, lower doses (5 or 15 μg) induced higher magnitudes of CD4^+^ T cells than higher doses (50 or 150 μg), which could be detected up to 182 days after vaccination. Recombinant Ag85B/ESAT-6/Rv2660c fusion protein formulated with IC31 (H56:IC31) also induced polyfunctional CD4^+^ T cells in both previously BCG-immunized, Mtb-uninfected and Mtb-infected South African adults (76). Frequencies of these polyfunctional CD4^+^ T cells were higher in Mtb-infected than uninfected individuals and comprised the predominant proportion of the cytokine-producing cells 70 days after vaccination. These cells persisted, expressing markers of central memory cells, up to 210 days after immunization. As in the H4:IC31 study, a lower dose of H56:IC31 resulted in higher proportions of polyfunctional CD4^+^ T cells while in recipients of a high 50 μg H56 dose, CD4^+^ T cells expressing only IFN-γ predominated. Recombinant Ag85B/ESAT-6 formulated in IC31 (H1:IC31) induced H1-specific polyfunctional CD4^+^ T cells in Mtb-uninfected and infected South African adolescents which persisted at least 70 days in Mtb-infected and 224 days in Mtb-uninfected individuals (77), and in HIV-infected Tanzanian adults, which peaked at 70 days and persisted for 182 days after vaccination (78). Finally, a recombinant Mtb32A/Mtb39A fusion protein formulated in AS02_A_ (Mtb72F:AS02_A_) induced polyfunctional CD4^+^ T cells in Mtb-infected and previously BCG-immunized Swiss adults (79), a recombinant Mtb32A/Mtb39A modified to increase stability formulated in ASO1_B_ (M72:ASO1_B_) or in AS02_A_ (M72:AS02_A_) induced polyfunctional CD4^+^ T cells in BCG naïve, Mtb-uninfected Belgian adults (80), and M72 formulated in AS01_E_ (M72: AS01_E_) induced polyfunctional CD4^+^ T cells in Mtb-infected and uninfected South African adults and adolescents (81, 82), HIV-infected and uninfected Indian adults (83), and HIV-infected Swiss adults on anti-retroviral therapy (84). In the latter study, polyfunctional CD4^+^ T cell populations also co-expressed CD40L and persisted from 7 months to 3 years after immunization. Thus, adjuvanted recombinant protein TB vaccine candidates are good inducers of CD4^+^ polyfunctional T cell responses in humans.

### Viral Vector Vaccines Elicit Polyfunctional T Cells

Adenoviral TB vaccine candidates have been studied in a few trials of infants and adults (Tables 4 and 5). In a Phase I double-blinded randomized placebo-controlled trial of BCG-immunized, Mtb and HIV-uninfected South African adults, an Ad35 expressing Ag85A, Ag85B, and TB10.4 (AERAS-402), induced polyfunctional CD4^+^ T cells that constituted the predominant CD4^+^ T cell subset 28 days after immunization, but this subset was not detectable 182 days after immunization (85). AERAS-402 also induced polyfunctional CD4^+^ T cells in BCG-immunized, HIV-infected South African adults, which peaked 42 days after the first vaccination (86). By contrast, in a double-blinded randomized trial of healthy BCG-immunized HIV-uninfected infants performed in South Africa, Kenya, and Mozambique, AERAS-402 induced detectable but low level CD4^+^ polyfunctional T cell responses, at frequencies lower than had been observed in BCG-vaccinated adults in the afore mentioned study (68). Finally, an Ad5 expressing Ag85A vaccine candidate induced polyfunctional CD4^+^ T cell responses in both BCG naïve and previously BCG-vaccinated, Mtb and HIV-uninfected Canadian adults (87). Polyfunctional CD4^+^ T cells comprised the predominant subset of cytokine-producing T cells and peaked at 2–4 weeks after vaccination. Therefore, adenoviral TB vaccines induce polyfunctional CD4^+^ T cell responses in humans that do not persist as long as those induced by protein subunit TB vaccines.

The capacity for MVA expressing A85A (MVA85A) to elicit polyfunctional CD4^+^ T cells, as a boost to previous BCG vaccination, has been extensively evaluated in several studies of infants, children, adolescents, and adults (Tables 4 and 5). MVA85A induced polyfunctional CD4^+^ T cell responses in previously BCG-vaccinated Mtb- and HIV-uninfected South African adults (88), Mtb- and HIV-uninfected British adults (89, 90), Mtb-infected HIV-uninfected South African adults (91), Mtb-infected HIV-uninfected British adults (92), and Mtb- and HIV-uninfected infants (3, 69), young children (69) and adolescents (69). In these studies, polyfunctional CD4^+^ T cells represented the predominant subset of cytokine-producing T cells and persisted at least up to 168 days after vaccination. Moreover, in a follow-up study of the South Africa cohorts (69, 88, 91), Tameris et al. demonstrated that MVA85A induced polyfunctional CD4^+^ T cell responses persisted 3–5 years after immunization (70). Of note, these T cell responses were of lower magnitude in infants (3) than observed previously in adults (89). Thus, MVA85A is a potent inducer of polyfunctional CD4^+^ T cells responses, which persist for 3–5 years after vaccination.

## Correlation of Polyfunctional T Cell Responses and Risk of TB in Humans

Understandably little is known about the correlation of polyfunctional CD4^+^ T cells and risk of TB, although two infant vaccine trials have evaluated the relationship of vaccine-induced polyfunctional CD4^+^ T cells and subsequent development of TB. Kagina et al. performed a subanalysis of a large trial evaluating the immunogenicity of BCG (Japanese) delivered i.d. vs s.c. in South African infants (62). Infants who developed TB over the 2-year follow-up period were compared to healthy, Mtb exposed, and healthy Mtb unexposed infants. There was no correlation between the magnitude of polyfunctional CD4^+^ T cells and subsequent development of TB during 2 years of follow-up. More recently, Tameris et al. conducted a double-blind Phase IIb trial in South African infants evaluating the immunogenicity and efficacy of MVA85A as a boost after BCG prime at birth, compared with a placebo (3). Although MVA85A-vaccinated infants developed polyfunctional Ag85A-specific CD4^+^ T cell responses, albeit at lower magnitudes than typically observed in adults, the MVA85A boost provided no additional efficacy against development of TB over BCG alone. By contrast, the majority of mouse TB studies evaluating polyfunctional CD4^+^ T cells and vaccine-induced protection have noted a correlative relationship. This discrepancy may reflect differences between the mouse TB vaccine model and vaccine-induced protection from human disease, publication bias, or, in the case of the MVA85A study, possibly induction of inadequate levels of polyfunctional CD4^+^ T cells required for protection. Regarding the latter, Fletcher et al. found that immune activation at the time of MVA85A or placebo vaccination was associated with risk of developing TB, which in turn may be associated with poor BCG vaccine take, i.e., lower frequencies of BCG-specific IFN-γ-expressing cells (93). These data suggest that additional factors over and above the functionality of T cells play a role in protective immunity.

Discrepancies between some mouse TB vaccine studies and others, and between mouse and human TB vaccine studies, could reflect that a correlate of protection may be specific for each particular vaccine platform or may vary for different TB antigens. In this regard, standardization of vaccine study protocols and harmonization between animal and human studies could improve the ability to discern correlates of protection. In addition, discrepant results among animal model and human studies of polyfunctional CD4^+^ T cells, in general, could reflect that frequencies of polyfunctional CD4^+^ T cells correlate with a more accurate, yet-to-be determined correlate of protection. In this regard, mouse studies suggest that total IL-2-producing CD4^+^ T cells may be the best correlate of protection against Mtb infection (38, 41) and in humans, IL-2-producing CD4^+^ T cells are associated with successful containment of Mtb infection in persons with LTBI (13, 14). Conversely, loss of IL-2 expression by IFN-γ and/or TNF-expressing Th1 cells is likely indicative of greater T cell differentiation, typically observed in scenarios of high bacterial loads, such as TB disease (13, 14), or following delivery of high doses of vaccine antigens (76). This is consistent with the linear differentiation model of CD4^+^ T cells derived primarily from acute and chronic viral infections (22). In this model, IL-2-production reflects central memory T cells in early stages of differentiation, with capacity for long-lived memory and proliferation. Loss of IL-2 expression thus reflects more advanced differentiation of CD4^+^ T cells, such as effector memory or terminal effector cells. This is further supported by a recent study of CD4^+^ T cell responses to Ag85B and ESAT-6 in mice and humans, which showed that Ag85B-specific T cells were significantly less differentiated than ESAT-6-specific T cells, which correlated with lack of persistent Ag85B antigen expression and persistent ESAT-6 expression by the bacterium during chronic infection in the mouse model (94). Distinct attributes observed between these T cell subsets were higher proportions of polyfunctional and lower proportions of IL-2-expressing ESAT-6-specific T cells, when compared with Ag85B-specific T cells.

## Conclusion and Perspectives

BCG, as well as novel TB vaccine candidates, induce polyfunctional CD4^+^ T cells in both animal models and humans. These cells possess important functional attributes that may potentially play a role in vaccine-mediated protection including long-lived memory function, persistence in the vaccinated host, and at least in animal models, ability to traffic to and persist in the lung. In the mouse model, the magnitude of vaccine-induced polyfunctional CD4^+^ T cells often correlates with vaccine-induced protection, making polyfunctional T cells a good candidate for a mechanistic correlate of protection. However, definitive evidence that it is in fact the co-expression of IFN-γ, TNF-α, and IL-2 by these T cells, rather than another functional or phenotypic attribute, that mediates host defense against Mtb remains lacking and would require sophisticated knock-down or adoptive transfer experiments. Some mouse studies, and of note, two human infant studies do not support polyfunctional CD4^+^ T cells as correlate of protection. Moreover, because these studies focus mainly and often exclusively on defining polyfunctional CD4^+^ T cells, it is certainly possible that a stronger immunologic correlate was present and not measured. We conclude that induction of polyfunctional T cells is certainly not sufficient and may not even be necessary to mediate protective immunity against Mtb and speculate that the production of multiple pro-inflammatory cytokines by T cells may reflect properties of T cells that may not necessarily be required for protection. Other functional attributes, such as additional effector functions, the differentiation state, tissue homing potential, long-term survival capacity of the T cell, or their ability to recognize the Mtb-infected cell may be equally or more important to promote protection. It is also possible that the induction of polyfunctional CD4^+^ T cells may be dependent upon the particular antigen or adjuvant utilized. Thus, a correlate of protection for TB vaccine development remains elusive. We propose that studies of protective immunity against Mtb should reach well beyond the measurement of IFNγ, TNF, and IL-2 by investigating other functions, phenotypes and correlates of immunity. Further, in light of the lack of direct causal association between T cell polyfunctionality and protective immunity, care should be taken not to bias studies in favor of the hypothesis that polyfunctional cells are indeed the mediators of protection. The definition of correlates of protection may benefit from standardization of animal TB vaccine studies and harmonization of these protocols with human trials. Finally, future studies should address the full spectrum of CD4^+^ T cell flavors and colors within the context of more complete immunological signatures of protection, including for example, additional phenotypic and functional attributes of CD4^+^ T cells, such as IL-17 production, and other major cells, such as classically restricted CD8^+^ T cells, donor unrestricted T cells, and NK cells, as well as B cells.

## Author Contributions

DAL performed the literature search and designed the tables. DAL and TS outlined and wrote the manuscript. DAL, DML, and TS discussed and edited the manuscript.

## Conflict of Interest Statement

The authors declare that the research was conducted in the absence of any commercial or financial relationships that could be construed as a potential conflict of interest.
